# Lithium, Tin(II), and Zinc Amino-Boryloxy Complexes: Synthesis and Characterization

**DOI:** 10.1021/acs.inorgchem.2c03108

**Published:** 2023-01-28

**Authors:** Andrew J. Straiton, Claire L. McMullin, Gabriele Kociok- Köhn, Catherine L. Lyall, James D. Parish, Andrew L. Johnson

**Affiliations:** † Department of Chemistry, University of Bath, Claverton Down BA2 7AY, U.K.; ‡ Material and Chemical Characterisation Facility, University of Bath, Claverton Down BA2 7AY, U.K.; § Infineum UK Ltd., Milton Hill Business and Technology Centre, Abingdon OX13 6BB, Oxfordshire, U.K.

## Abstract

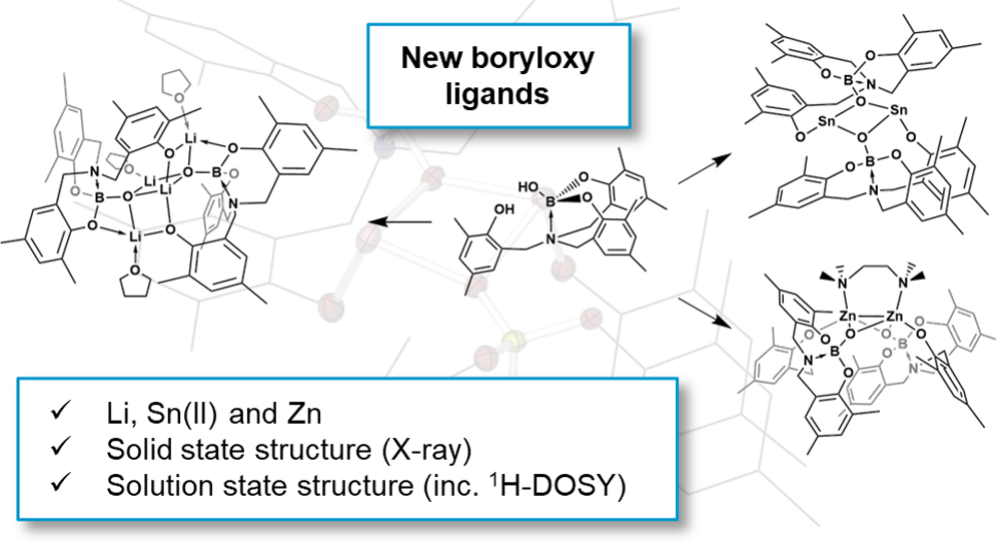

Analogous to the ubiquitous alkoxide ligand, metal boroxide and boryloxy complexes are an underexplored class of hard anionic O^–^ ligand. A new series of amine-stabilized Li, Sn(II), and Zn boryloxy complexes, comprising electron-rich tetrahedral boron centers have been synthesized and characterized. All complexes have been characterized by one-dimensional (1D), two-dimensional (2D), and DOSY NMR, which are consistent with the solid-state structures unambiguously determined via single-crystal X-ray diffraction. Electron-rich μ^2^- (Sn and Zn) and μ^3^- (Li) boryloxy binding modes are observed. Compounds **6**–**9** are the first complexes of this class, with the chelating bis- and tris-phenol ligands providing a scaffold that can be easily functionalized and provides access to the boronic acid pro-ligand, hence allowing facile direct synthesis of the resulting compounds. Computational quantum chemical studies suggest a significant enhancement of the π-donor ability of the amine-stabilized boryloxy ligand because of electron donation from the amine functionality into the p-orbital of the boron atom.

## Abstract 2

Here we present the synthesis and solution and solid-state characterization of a new and previously unexplored class of Lewis-base-stabilized metal boryloxy ligated complexes of Li, Sn(II), and Zn. These new boron-based oxygen ligands provide a new and synthetically versatile route to electronically rich boron-based oxygen ligands and their complexes, which have been studied via 1D, 2D, and DOSY NMR alongside single-crystal X-ray diffraction studies and density functional calculations.

## Introduction

Anionic oxygen-based ligands, such as alkoxides, aryloxides, polyphenols, and salens, are ubiquitous across 21st-century chemistry.^1^ Lone pairs on the oxygen atoms of these ligands are generally available to donate to suitable orbitals on the metal fragment giving alkoxide ligands the potential to donate 2σ + 4π electrons to a metal center. As such, this ligand class plays a significant role in the coordination chemistry of electron-deficient metal centers (i.e., early transition-metal elements, lanthanides) and is much less common in the chemistry of the late d-block metals. This electronic flexibility, combined with the ability to sterically modify and functionalize alkoxide ligands has made it the focus of significant research for a diverse range of applications, such as catalysis^2^ and as precursors to materials.^3^

**Figure 1:**
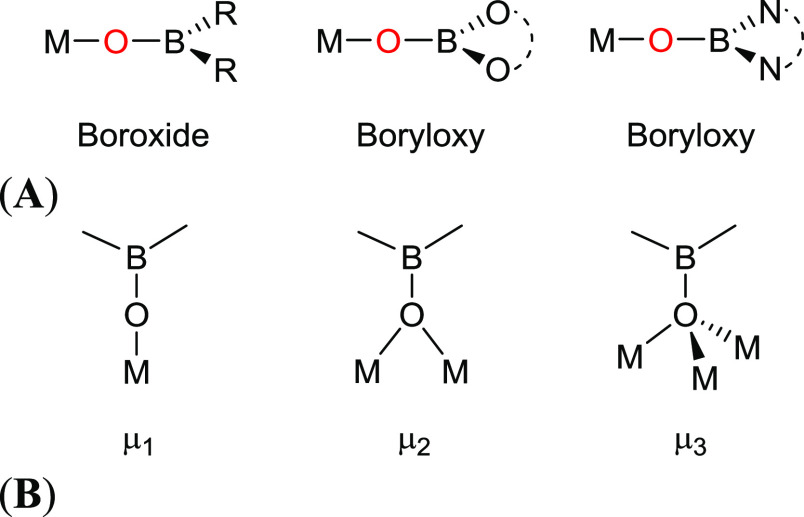
Types of boroxide and boryloxy ligand systems (A), and the different boroxide coordination modes reported (B).^4^

In contrast, boroxide, [R_2_BO]^−^, and boryloxy ligands, [(RO)_2_BO]^−^ and [(R_2_N)_2_BO]^−^ (Figure 1), which have more recently been explored as a new class of oxygen-based ligands, are generally considered to be electron-deficient variants of alkoxide systems. The O-atom lone pairs are of the correct symmetry to combine with the empty 2p-orbital on the boron atom, resulting in an overall reduction in the electron density available for donation to a metal center.^4^ These ligands have been shown to possess very similar coordination modes to alkoxides with terminal (μ^1^) and bridging (μ^2^) modes having been observed in the solid state, alongside the less common face capping (μ^3^) coordination mode.^5,6^ While computational studies suggest that M-OBR_2_ bonding is principally ionic in character,^7^ the net result is that the boroxide ligands behave as weak π donor ligands compared to an alkoxide. Metals coordinated to such ligands can therefore be considered electron-deficient compared with a structurally similar alkoxide complex.

**Figure 2:**
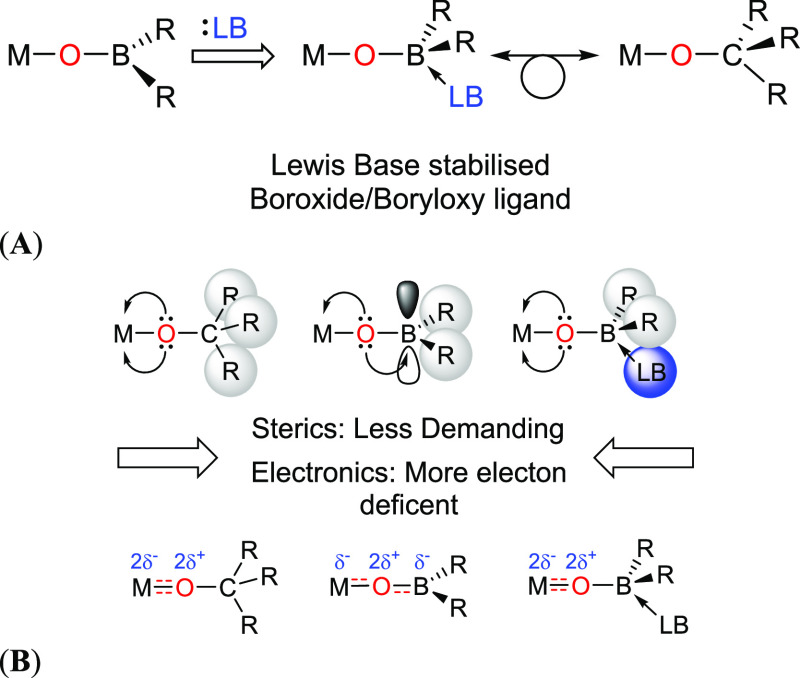
Schematic highlighting the formation of a Lewis-base-stabilized boroxide/boryloxy ligand system (A) and the steric and electronic differences between alkoxide, boroxide, and Lewis-base (LB)-stabilized boroxide/boryloxy ligand (B).

One possible strategy to reverse this electronic perturbation and render boroxide and boryloxy ligands stronger π-donors is to inhibit O–B π-back bonding with the inclusion of a Lewis basic moiety as part of the ligand scaffold, capable of interacting with empty 2p-orbital on the boron atom, thus making the Lewis-base-stabilized boroxide (or boyloxy) both sterically and electronically similar to the iso-electronic alkoxide ligand (Figure 2).

**Figure 3:**
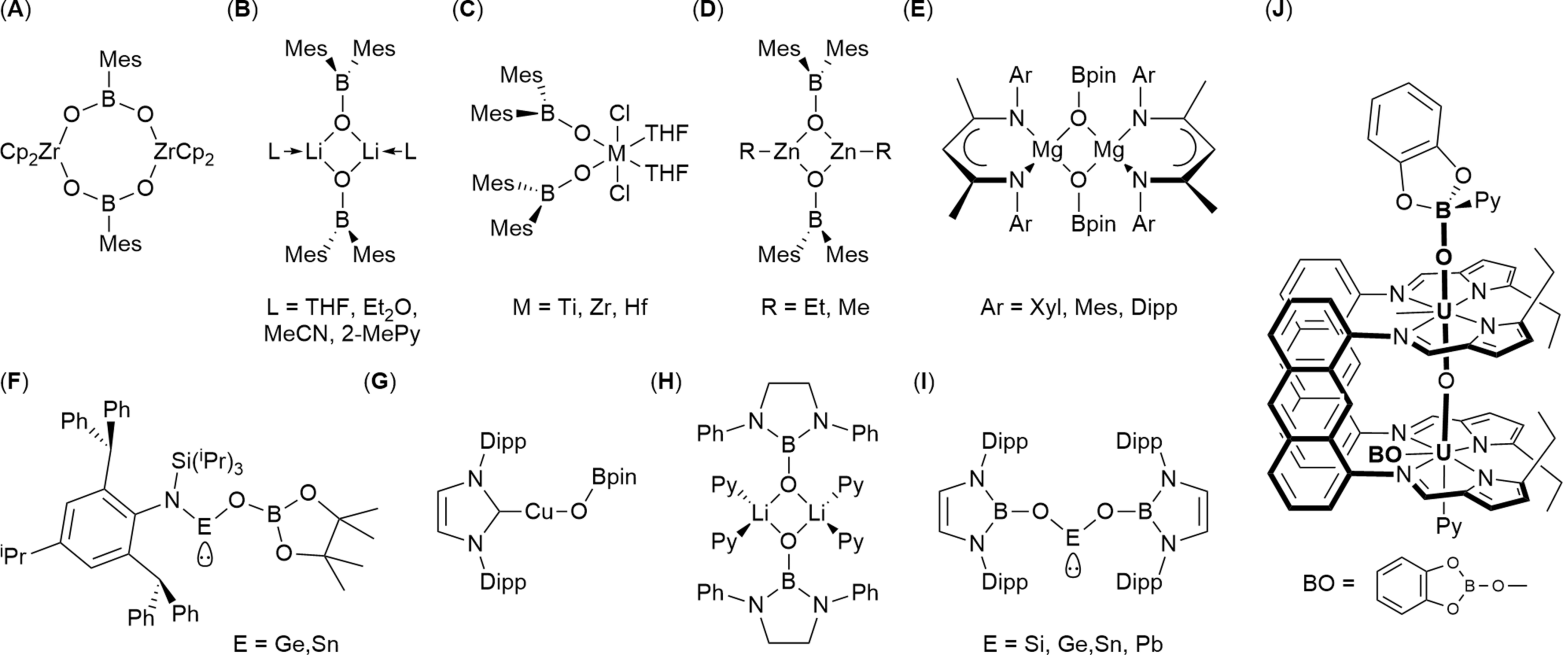
Selected examples of metal boroxide and boryloxy systems.

Given the vast quantity of research conducted on metal alkoxides, it is perhaps surprising that there are relatively few boroxide and boryloxy systems known in the literature. As such, our understanding of the chemistry of these systems is not fully developed. Examples have been reported based upon both boronic acids, {RB(OH)_2_} (Figure 3A),^8,9^ and borinic acids {R_2_BOH}. In addition to being more electron-poor than their boronic acid counterparts, borinic acids can also feature two bulky substituents (e.g., {Mes_2_BOH}), sterically stabilizing the resulting metal complex. Examples from across the periodic table are known, including, but not limited to, alkali metals,^10−13^ early transition metals,^14−16^ and group 12^5,17^ and group 14^18−20^ metals. (Figure 3B–D). A 2016 review by Coles’ provides the most up to date overview of these systems.^4^

A number of boroxide complexes derived from pinacol borate exist, featuring groups 2,^7,21−23^13,^24^ 14,^25^ and a number of transition metals^26−28^ (Figure 3E–G). These systems are more electron-rich at the boron center compared to boronic and borinic acid derivatives due to donation into the vacant p-orbital by the additional oxygen atoms of the {Pin} ligand. Notably, however, the examples reported here arise from reactions with HBpin, not via metathesis reactions with the parent pro-ligand or boroxide salt as is routinely the case for the boronic and borinic acid derivatives. Further examples feature N-heterocyclic boryloxy ligands, analogous to NHCs, coordinated to Group 1,^29^ 14^30^ (Figure 3H,I), and lanthanide centers.^31^

While both metal boroxides and metal boryloxy complexes, synthesized as intermediates in catalytic cycles, have been reported, the number is insignificant compared to the number of metal alkoxides reported. A search of the literature reveals one example of a Lewis-base-stabilized tetrahedral boron center, a pyridine catecholboroxy uranium complex (Figure 3J).^32^ In this example, the vacant p-orbital on the boron center is stabilized via a dative bond from the pyridine, resulting in a more electron-rich tetrahedral boron center, as depicted in Figure 2. The discrepancy between the bonding in the three- and four-coordinate boron centers on this complex is significant (B–O = 1.40 Å, O–U = 2.09 Å,^4^ B–O = 1.31 Å, O–U = 2.22 Å^3^); however, this compound is isolated in very small quantities and decomposes with the loss of the boroxide ligands.

**Scheme 1:**
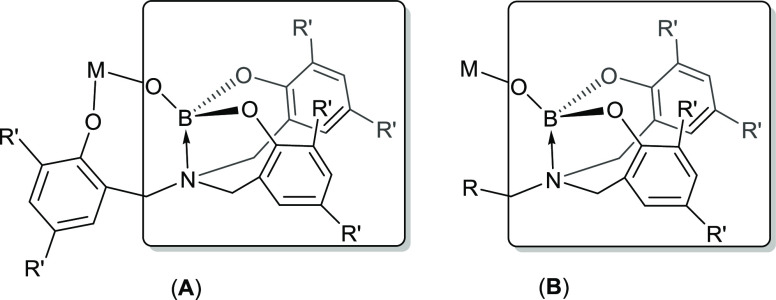
Amino-tris-phenoxy-boryloxy (**A**) and Amino-bis-phenoxy-boryloxy Ligands (**B**) with Their Core Amine-Stabilized Boryloxy Unit Highlighted.

As part of an effort to devise new ligand architectures, and to synthesize more electron-rich metal boryloxy species than those previously reported, we present a series of compounds derived from electron-rich borate ester ligands, featuring a stable tetrahedral boron center. Reactions of polydentate amino-phenols with B(OH)_3_ afford amine-stabilized boronic acid derivatives, systems suitable for onward reaction as pro-ligands i.e., amino-tris-phenoxy-boryloxy (**A**) and amino-bis-phenoxy-boryloxy ligands (**B**), both of which contain the same amine-stabilized boryloxy fragment as shown in Scheme 1, capable of coordinating to, and stabilizing metal centers.

In a preliminary study, reaction of these new pro-ligands with selected metal reagents has enabled the synthesis of their Li, Sn(II), and Zn complexes, respectively. The structural motifs in these compounds have been analyzed using single-crystal X-ray diffraction and multinuclear NMR spectroscopy. A series of ^1^H-DOSY NMR experiments have been conducted to further understand the solution-state structures of these complexes, using a recently developed technique for molecular weight estimation. The electronic nature of the ligands has also been explored by density functional theory. We have also attempted to quantify the donor capabilities of the amine-boryloxy pro-ligands in comparison with other oxy using density functional calculations. To the best of our knowledge, these systems represent the first example of a new class of tunable boryloxy systems.

## Results and Discussion

### Synthesis of Boronic Acid-Derived Pre-Ligands **1–4**

**Scheme 2:**
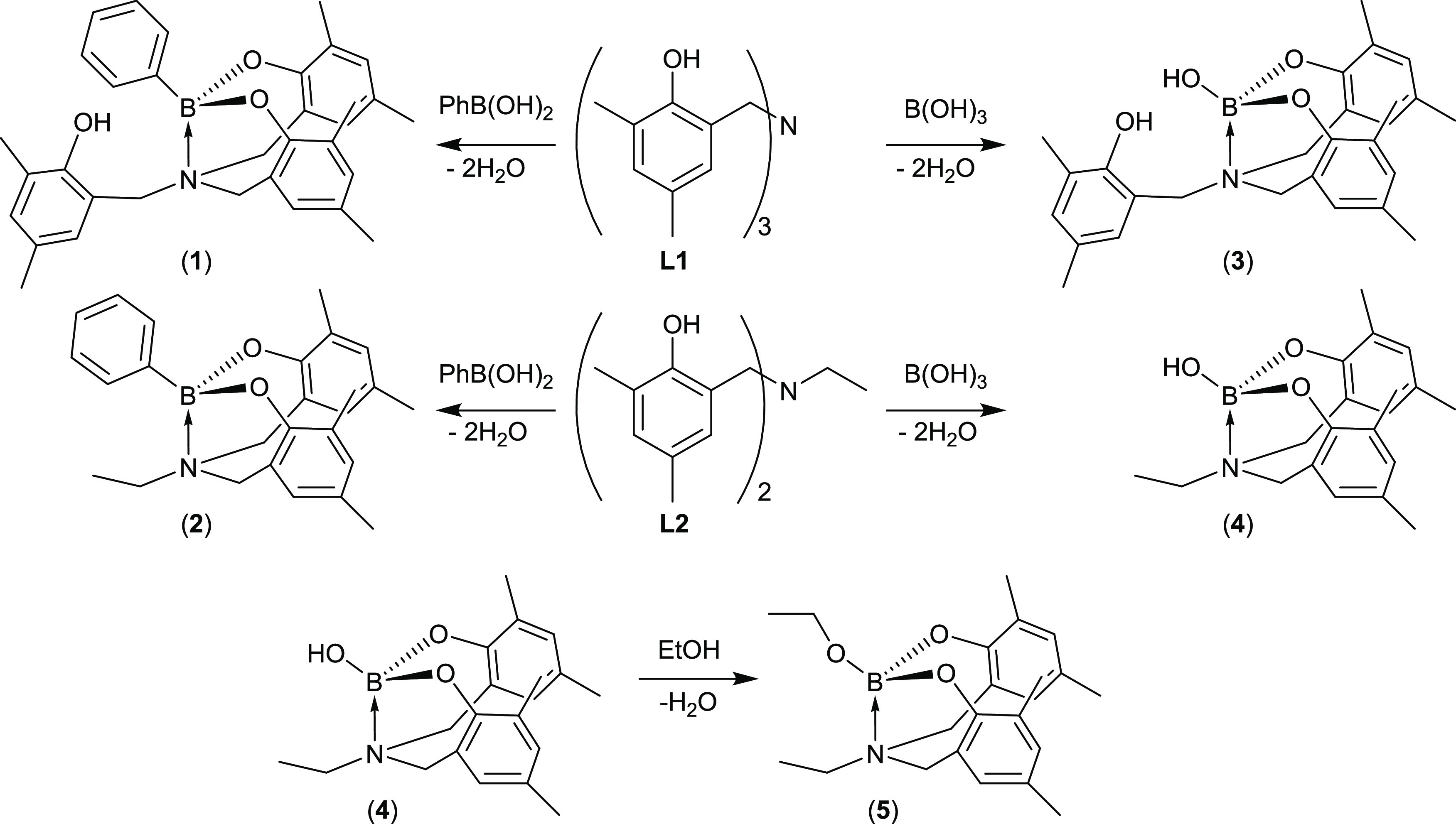
Formation of Complexes **1**–**5**

The phenolic ligands, **L1** and **L2**, formed via Mannich condensation reactions,^33^ were reacted stoichiometrically with phenylboronic acid in tetrahydrofuran (THF) under ambient conditions to yield, upon recrystallization from dichloromethane (DCM)/hexane and chloroform, respectively, complexes **1** and **2** as colorless crystals (Scheme 2). Complexes were characterized using multinuclear NMR, high-resolution mass spectrometry, and single-crystal X-ray diffraction, with samples confirmed to be analytically pure by elemental analysis.

The ^1^H NMR spectra of **1** and **2** in *d*_2_-DCM display three aromatic resonances representative of the phenylboronic acid groups alongside two further resonances in the aromatic region, which correspond to the aromatic protons found on the ligand. These two resonances (δ = 6.56, 6.97 for **1**, δ = 6.62, 6.96 for **2**) correspond to the protons found in the meta-position of the aromatic ring, the two aromatic rings bound to the boron center appearing in equivalent chemical environments in both cases. The methylene group in the two positions of the aromatic ring appears as two doublets with *J* = 15 Hz, indicative of ^2^*J* coupling, demonstrating the two protons are diastereotopic. In the case of compound **1**, the unbound phenol system is observed in a different chemical environment. Both of these observations are indicative of a tightly bound complex, with no exchange occurring on the NMR timescale. The ^11^B spectra of **1** and **2** display single resonances at δ = 4.29 and 4.10 ppm, respectively, significantly upfield of that observed for phenylboronic acid (δ = 29.5 ppm), indicative of the greatly increased electron density on the boron center.

**Figure 4:**
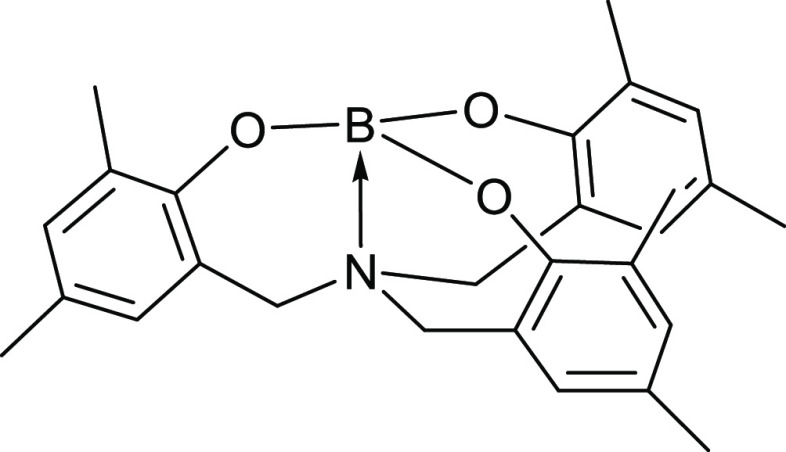
Boratrane complex obtained upon reaction of **L1** with B(OMe)_3_ under inert/anhydrous conditions.^35^

The reaction of **L1** and **L2**, respectively, with B(OH)_3_, again under ambient conditions, yielded **3** and **4** (Scheme 2). The reaction of **L1** with boron and other group 13 reagents is not unprecedented;^34,35^ however, rather than the boratrane complex (Figure 4) that is obtained upon the reaction of **L1** with B(OMe)_3_ in inert/anhydrous conditions, the alternative, stable hydroxy product, **3**, was obtained. Compound **3** could therefore also be obtained via the inert synthesis and subsequent hydrolysis of the boratrane complex, yet the procedure reported within this work is a significantly more facile route to **3**. While it was possible to obtain colorless crystals of **3**, via recrystallization in toluene, successive attempts to recrystallize **4** for single-crystal studies were unsuccessful; however, ^1^H, ^11^B, and ^13^C{^1^H} NMR spectra showed the formation of a single, clean product. It was possible to obtain crystals of the analogous ethyl ester, **5** (see SI: Figure S1), upon dissolving **4** in ethanol, demonstrating the initial presence of **4**.

The ^1^H NMR spectra of compounds **3** and **4** are comparable to those obtained for **1** and **2**. Compound **3** displays two sets of aromatic environments in a 2:1 ratio, corresponding to the bound and free phenolic groups. This is indicative of strong coordination to the boron center, with no exchange between the phenol groups in solution on the NMR timescale. In the case of **4**, a single set of aromatic resonances is observed, with a multiplet resonance comprising overlapping doublets observed for the diastereotopic methylene protons. A single resonance was observed for each compound in the ^11^B NMR spectra at δ = 2.40 and 2.33 ppm, respectively. The upfield shift *cf.* complexes **1** and **2** is indicative of increased electron density at the boron center, resulting from the replacement of the phenyl group with the more electron-donating boronic acid group.

### Molecular Structures of Compounds **1**–**3**

**Figure 5:**
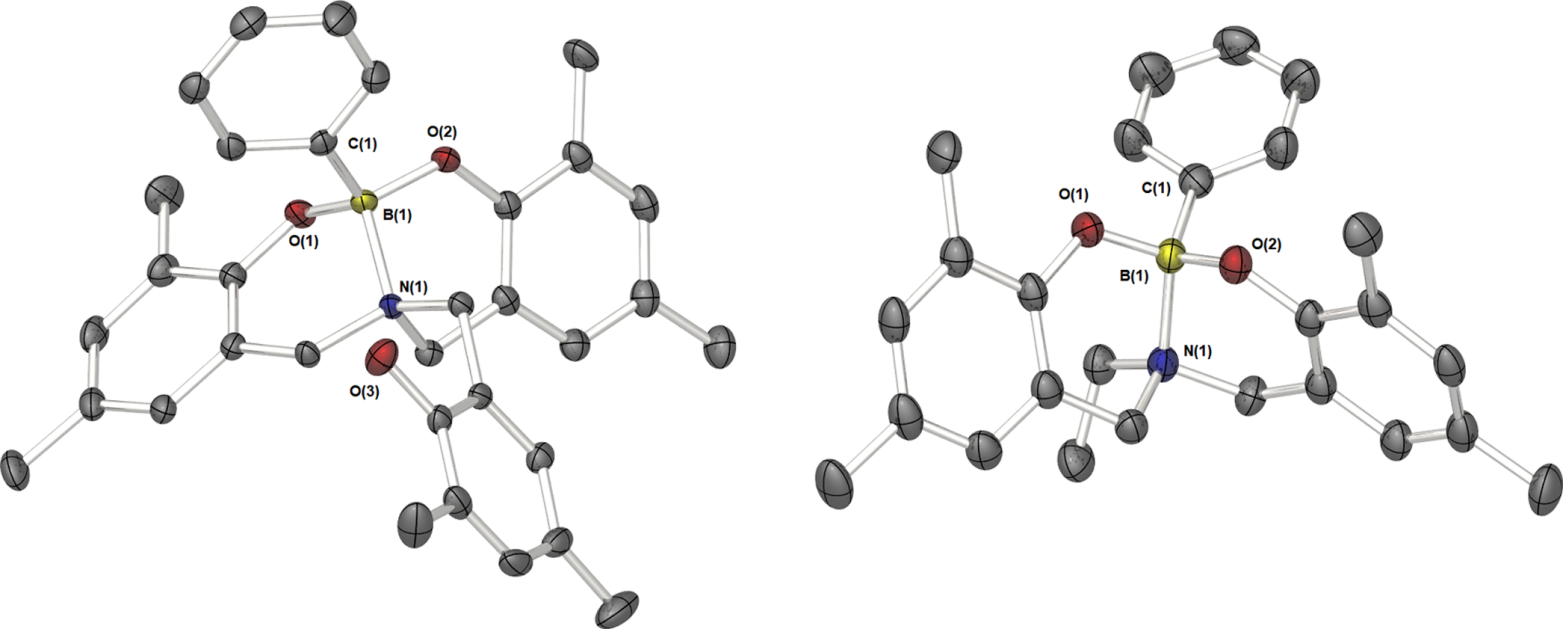
Molecular structures of **1** and **2**. Thermal ellipsoids are shown at 50% probability. Hydrogen atoms, the second molecule of compound **1**, and chloroform found in the unit cell of **2** have been omitted for clarity.

**Figure 6:**
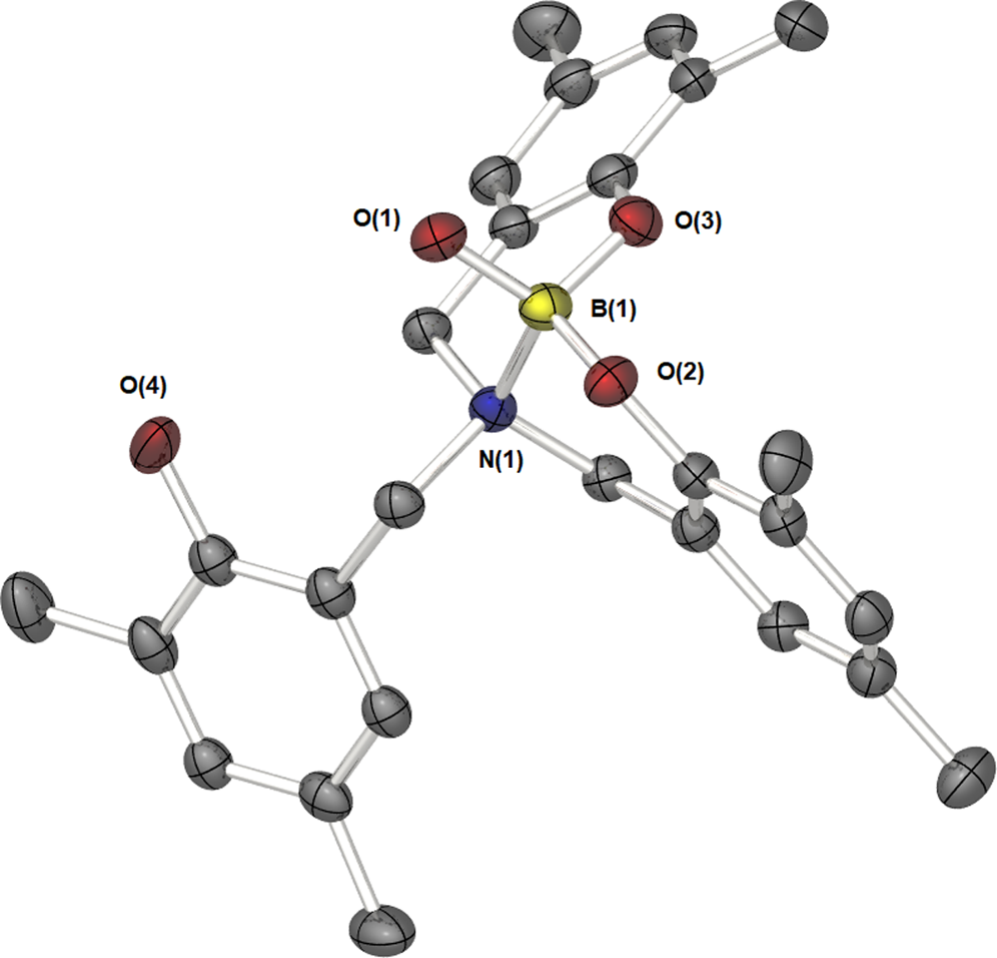
Molecular structure of the phenol-*N*-stabilized boronic acid, **3**. Thermal ellipsoids are shown at 50% probability. Hydrogen atoms, the second molecule present in the unit cell, and THF/toluene as solvent of crystallization have been omitted for clarity.

X-ray diffraction studies on single crystals of compounds **1**, **2**, and **3** unambiguously established their solid-state structures, as shown in Figure 5 (**1** and **2**) and Figure 6 (**3**). Selected bond lengths and angles are given in Table 1 (**1** and **2**) and Table 2 (**3**). Crystal and structure refinement data for compounds **1**, **2**, **3**, and **5** are presented in Table S2.

**Table 1:** Selected Bond Lengths and Angles for Compounds **1** and **2**

	bond lengths (Å)	
	**1**	**2**
B(1)–O(1)	1.468(2)	1.397(4)
B(1)–O(2)	1.4486(19)	1.469(5)
B(1)–N(1)	1.6611(19)	1.768(5)
B(1)–C(1)	1.616(2)	1.579(5)

	bond angles (deg)	
	**1**	**2**
O(1)–B(1)–O(2)	109.29(12)	109.4(3)
O(1)–B(1)–N(1)	105.01(11)	110.0(3)
O(1)–B(1)–C(1)	112.95(12)	105.1(3)
N(1)–B(1)–C(1)	112.47(12)	112.5(3)

**Table 2:** Selected Bond Lengths and Angles from Compound **3**

bond lengths (Å)		bond angles (deg)	
B(1)–O(1)	1.431(3)	O(1)–B(1)–O(2)	111.48(17)
B(1)–O(2)	1.443(3)	O(1)–B(1)–O(3)	111.82(17)
B(1)–O(3)	1.464(3)	O(1)–B(1)–N(1)	109.41(16)
B(1)–N(1)	1.659(3)	O(2)–B(1)–O(3)	110.84(17)
		O(2)–B(1)–N(1)	107.38(16)
		O(3)–B(1)–N(1)	105.64(16)

Compounds **1** and **2** crystallize in the space groups *P*1̅ and *Pca*2_1_, respectively, with two molecules found in the unit cell of compound **2**. While not crystallographically identical, the differences in the two molecules are inconsequential. Both compounds have analogous {PhBNO_2_} cores, with the boron center bound by the two phenolic oxygens and an additional nitrogen center, resulting in the formation of two fused six-membered rings. The dative N–B interaction, in which the donor N atom donates into the otherwise vacant boron p-orbital is the cause of the tetrahedral geometry (τ_4_′ = 0.95 and 0.95),^36^ and is observed clearly in the relevant bond lengths in both compounds. In compound **1**, no interaction is observed between the boron center and the pendant hydroxybenzyl group O(3).

Compound **3**, which has a molecular structure analogous to compound **1**, crystallizes in the space group *C*2/*c*, with two whole molecules found in the unit cell alongside two molecules of THF and one molecule of toluene as solvent of crystallization (Figure 6). While crystallographically inequivalent, any differences are inconsequential and likely the result of crystal packing effects. The boron center possesses a tetrahedral coordination geometry (τ_4_′ = 0.97) and is at the center of the {BNO_3_} core. The presence of a boronic acid OH group (O1), replacing the phenyl ring found in **1**, has no significant effect on the B–O or B–N bond lengths compared to compounds **1** and **2**. The B–O bond lengths are all analogous to those reported in the similar bis(napthoxypyridine) complex.^37^ No interaction is observed between the boron center and the pendant hydroxybenzyl group O(4).

Compound **5**, the ethyl ester of compound **4**, crystallizes from ethanol in the space group *P*21/*c* (Figure S1). Selected bond lengths and angles are given in Table S3. The tetrahedral boron atom (τ_4_′ = 0.96) is at the center of the {BNO_3_} core. The bond lengths of the boron center are similar to those found in **3**. The exception is the B(1)–O(1) bond, which is determined to be marginally shorter, an effect likely a result of the hyperconjugation arising from the ethyl ester, which results in a more electron-rich oxygen center.

### Synthesis and Characterization of Metal Boryloxy Complexes **6–9**

#### Synthesis of Li Complex

**Scheme 3:**
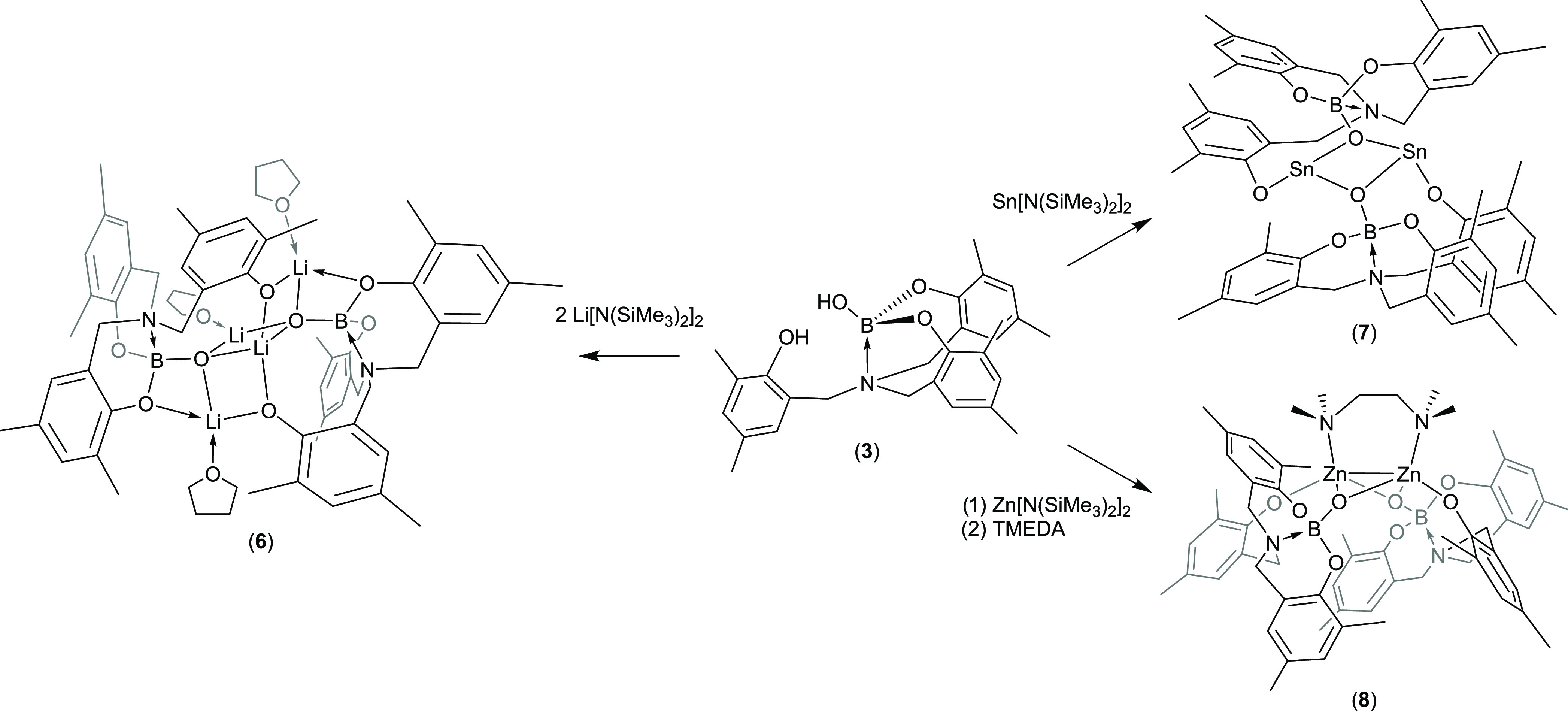
Formation of Complexes **6**, **7**, and **8**

The reaction of compound **3** with 2 equiv of [Li{N(SiMe_3_)_2_}] resulted in the formation of compound **6** (Scheme 3). Initial reaction and subsequent recrystallization in THF at −28 °C afforded **6** as colorless crystals in good yield (68%).

Initial ^1^H and ^7^Li NMR studies, undertaken in C_6_D_6_, suggested a range of products had formed; however, spectra obtained in *d*_8_-THF demonstrated that a single clean product had formed. The ^1^H NMR (Figure S3), and ^13^C{^1^H} (Figure S4) spectra show three sets of resonances corresponding to the three phenolic ring systems, full assignment of which is possible with the use of two-dimensional (2D)-NMR. The six diastereotopic methylene protons are again found in distinct environments, appearing at δ = 3.29/5.03, 3.38/5.08, and 3.73/4.07 ppm.

**Figure 7:**
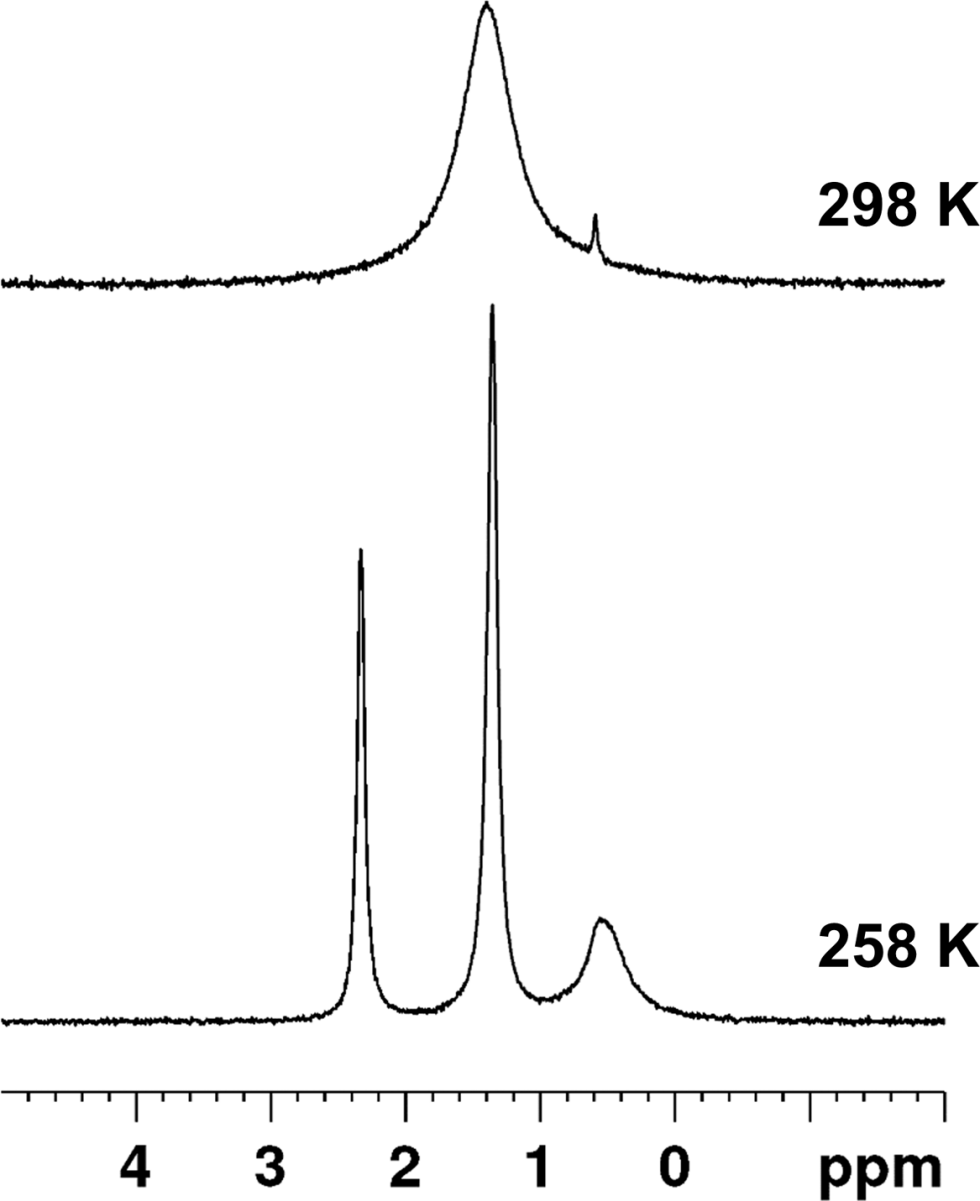
^7^Li-NMR spectra of complex **6** in *d*_8_-THF at 298 and 258 K.

The ^11^B NMR spectrum contains a single peak at δ = 3.04 ppm, suggesting the boron is slightly deshielded upon complexation, reflective of the electron-poor lithium center in the complex. At room temperature, the ^7^Li spectrum contains a single resonance at δ = 1.40 ppm; however, upon cooling to 258 K, three resonances are observed at δ = 2.33, 1.36, and 0.56 ppm, which integrate with a 1:2:1 ratio (Figure 7), an observation consistent with the three lithium environments observed in the molecular structure. The reaction of **4** with 1 equiv of [Li{N(SiMe_3_)_2_}] in THF gave an insoluble white product that remained insoluble upon addition of either pyridine or the donor base *N*,*N*,*N*′,*N*′-tetramethylethylenediamine (TMEDA) as additional donor ligands.

#### Synthesis of Sn(II) Complex

Having identified the structure of **6**, the pro-ligand system **3** was subsequently reacted with 1 equiv of [Sn{N(SiMe_3_)_2_}_2_] (Scheme 3) in an attempt to afford an analogous main-group system. Initial reaction in toluene gave an insoluble white powder; however, crystallization from THF at 4 °C yielded **7** as colorless crystals.

The molecular structure (vide infra) shows the formation of a molecular dimer, with each molecular unit found to be equivalent. This results in a ^1^H NMR spectrum similar to that of **6**, with three distinct sets of resonances observed for each phenolic ring system (Figure S6). 2D-NMR experiments again allow complete assignment (see SI). Six distinctive doublet resonances, assigned to the six diastereotopic methylene protons appear at δ = 3.10/5.84, 3.19/4.30, and 3.29/5.91 ppm. While conventional wisdom would predict the distinctive pair at δ =3.19/4.30 to correspond to the Sn-bound phenol group, the observed magnitude of the chemical shift is instead found to correlate more closely with the position in space within the molecular structure. The pair of resonances at δ = 3.19/4.30 ppm corresponds to a B-bound ring, while the doublets found at δ = 3.29/5.91 ppm correspond to the methylene group on the lariat Sn phenoxide ring. While this initially appears counter-intuitive, study of the molecular structure shows the protons assigned to the doublets at δ = 5.84 and 5.91 ppm to be in chemically different yet spatially similar environments, with both being close in space to the Sn metal center. In contrast, while the proton assigned to the doublet at δ = 4.30 ppm appears to be in a chemical environment similar to that of the proton assigned to the resonance at δ = 5.91, the difference in the spatial environment of these protons results in the discrepancy in chemical shift.

In contrast, the three phenolic carbons are found at δ = 149.1, 149.6, and 157.9 ppm in the ^13^C{^1^H} spectrum (Figure S7), values which directly correlate to their chemical environment, the most deshielded being that of the Sn-bound phenolic group. The ^11^B NMR spectrum shows a single resonance at δ = 2.63, again suggesting minimal change in the electron density of the boron center. The ^119^Sn spectrum has a single resonance at δ = −412.3, a value significantly upfield of analogous three-coordinate Sn phenoxide systems reported previously,^38^ suggestive of a more shielded Sn center.

#### Synthesis of Zn Complexes

The stoichiometric reaction of **3** with [Zn{N(SiMe_3_)_2_}_2_] in THF resulted in the formation of an insoluble white precipitate. However, the addition of 1 equiv of the donor base TMEDA resulted in redissolution of the precipitate (Scheme 3). Removal of the THF solvent and recrystallization from toluene at room temperature gave the product as colorless crystals. The addition of 1 equiv of TMEDA per Zn, was presumed to result in the formation of the putative complex [(TMEDA)Zn{OBN-O}] [OBN-O = (OB{OC_6_Me_2_H_2_CH_2_}_2_N-{CH_2_C_6_Me_2_H_2_O})]. However, inspection of the NMR spectra of **8** reveals a 1:2 ratio of TMEDA to boryloxy ligand in the final complex, a feature confirmed in the solid-state molecular structure (vide infra).

Initially, ^1^H and ^13^C{^1^H} NMR spectra of **8**, obtained in C_6_D_6_, appeared to suggest the presence of multiple products, and use of 2D experiments established that this instead arose from each of the six phenolic rings being in distinct chemical environments, analogous to the molecular structure (vide infra) where no molecular symmetry is observed. This results in the observation of 42 ^1^H environments (Figure S9) and 60 ^13^C environments (Figure S10), full assignment of which is possible (Table S1) using 2D NMR and differentiation of the ring systems via comparison with the molecular structure. This is exemplified again by the methylene group of the ligand, which is found in 12 distinct ^1^H environments, arising from six pairs of diastereotopic protons, across three phenol rings on each of the two ligands. This is indicative of a highly ordered and rigid molecular structure afforded via an additional lariat phenol group. ^1^H and ^13^C{^1^H} NMR spectra obtained at 328 K showed a retention of these distinct environments at elevated temperatures, indicative of a very strongly bound and rigid complex. The ^11^B spectrum of **8** shows one resonance at δ = 2.74 ppm, demonstrating no significant alteration in the electron density of the boron center upon complexation.

**Scheme 4:**
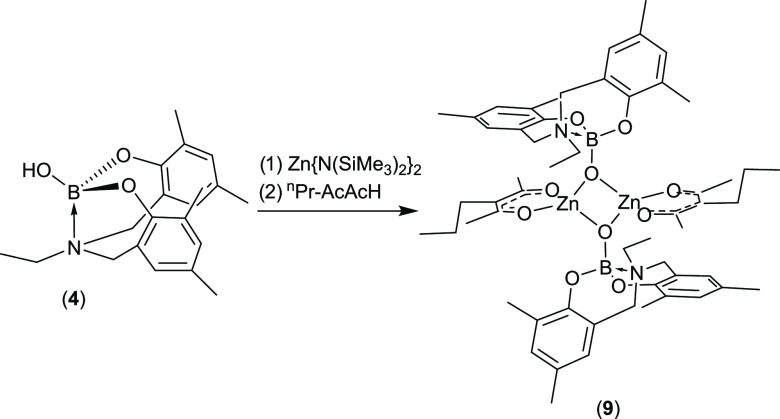
Formation of Complex **9**

The stoichiometric 1:1:1 reaction of **4** with [Zn{N(SiMe_3_)_2_}_2_] and *^n^*Pr-AcAcH resulted in the formation of complex **9** as pale-yellow crystals upon recrystallization from a mixture of DCM and hexane at 4 °C (Scheme 4). The ^1^H NMR spectrum of **9** in C_6_D_6_ (Figure S12) shows the presence of **4**, coordinated to the metal center alongside the *^n^*Pr-AcAc ligand in a 1:1 ratio. ^1^H DOSY NMR (vide infra) shows a single diffusion coefficient, consistent with the formation of a heteroleptic product.

Much like **4**, only one set of aromatic resonances are observed for the amino-tris-phenoxy-boryloxy ligand; however, in contrast to **4**, only a single broad resonance at δ = 3.60 ppm is observed for the methylene protons. A single set of resonances is observed for the {AcAc} ligand, with the α-CH_3_ groups appearing equivalent as a singlet at δ = 1.92 ppm. Analogous observations can be made by studying the ^13^C{^1^H} spectrum of **9** (Figure S13). The ^11^B NMR spectrum shows a single resonance at δ = 2.82 ppm, indicating only a very slight change in the shielding of the boron center upon complexation. The 2:1 reaction of **4** with [Zn{N(SiMe_3_)}_2_] was attempted; however, it did not prove possible to isolate a single clean product.

### ^1^H DOSY NMR Studies of Compounds **6–9**

To further understand the structures of compounds **6**–**9** in solution, ^1^H-DOSY NMR experiments were undertaken to determine both the hydrodynamic radius and approximate molecular weight of the compounds in solution. Alongside the use of the Stokes–Einstein equation to determine the hydrodynamic radius,^39^ external calibration curves were used in conjunction with normalized diffusion coefficients to allow empirical molecular weight determination. This methodology has been recently developed by Stalke et al.^40,41^ to probe the structure of a series of organolithium species^42−44^ and employed recently by us to study a collection of Zn and Sn(II) complexes.^45,46^ The solid-state hydrodynamic radii and calculated molecular weights are all derived from the molecular structures (vide infra). A summary of the results obtained is given in Table 3.

**Table 3:** Diffusion Coefficients, Hydrodynamic Radii Determined by ^1^H-DOSY NMR and X-ray Diffraction, Errors between the Measured Radii, Molecular Weights Determined by ^1^H DOSY NMR (MW_Det_), Calculated Molecular Weights (MW_Cal_), and Errors in MW between the Calculated and Observed Values for **6**–**9**

compound	solvent	*D* (×10^–10^ m^2^ s^–1^)	*R*_H_^a^	*R*_H_^b^	error (%)^c^	*D*_norm_ (×10^–10^ m^2^ s^–1^)	MW_Det_ (g mol^–1^)^d^	MW_Cal_ (g mol^–1^)	error (%)^a^
**6**	*d*_8_-THF	5.97	7.61	6.21	22	5.26	942	915/1131	3/–17
**7**	C_6_D_6_	5.08	6.70	5.79	14	5.04	1241^b^	1114	11
**8**	C_6_D_6_	5.03	6.77	5.97	13	5.02	1014	1130	–10
**9**	C_6_D_6_	5.05	6.74	5.90	14	4.99	1024	1096	–6.6

a As determined by DOSY.

b As determined by single-crystal X-ray diffraction studies.

c Error = (100 × (*R*_H, DOSY_ – *R*_H, X-ray_)/*R*_H, X-ray_) and (100 × (MW_det_ – MW_cal_)/MW_cal_).

d MW_det_ corrected for molecular weight as recommended by Kreyenschmidt et al.,^47^ with *X*_corr_ = 1.23.

The ^1^H-DOSY spectra of **6** (Figure S5), obtained in *d*_8_-THF, also show a single diffusion coefficient at 5.97 × 10^–10^ m^2^ s^–1^. This corresponds to an observed hydrodynamic radius of 7.61 Å and a determined molecular weight of 942 g mol^–1^. The ^1^H NMR spectrum shows resonances corresponding to coordinated and uncoordinated THF; however, the molecular mass determined here is within 3% of the expected mass in the absence of THF. In contrast to this, the observed hydrodynamic radius is significantly greater than that found in the crystal structure. Given the discrepancies in these values, it stands that the molecular dimer observed in the solid state is retained in solution—an observation supported by the VT ^7^Li NMR spectra that shows three distinct environments at 258 K. However, it is clear that the coordinated solvent is labile, and additional interactions with the solvent significantly convolute the interpretation of the ^1^H-DOSY NMR spectrum.

Analogous to compound **6**, the ^1^H-DOSY NMR spectrum of **7** (Figure S8) displays a single diffusion coefficient at 5.08 × 10^–10^ m^2^ s^–1^, correlating to a hydrodynamic radius of 6.70 Å, an error of 13%. Due to the increased density of the Sn center, an empirically determined correction factor is to account for this is applied to MW_det_.^47^ This results in a value for MW_det_ of 1241 g mol^–1^, an error of 11%. Both of these approaches suggest an increased molecular size compared to the solid state; however, both are less than the expected empirical error of ∼15% commonly reported for DOSY NMR studies. We believe that the molecular dimer remains in solution, with interactions with solvent molecules more likely the cause of any discrepancy as opposed to higher-order oligomer formation.^48,49^

As discussed previously, the ^1^H and ^13^C{^1^H} NMR spectra of **8** showed resonances corresponding to 2 equiv of the borate framework. This is an indication that the molecular dimer observed in the solid state is retained in solution, an observation supported by the ^1^H-DOSY NMR spectrum (Figure S11), which shows a single diffusion coefficient at 5.03 × 10^–10^ m^2^ s^–1^, and corresponds to a hydrodynamic radius of 6.77 Å, and a determined molecular weight of 1014 g mol^–1^ compared to expected values of 5.97 Å and 1130 g mol^–1^. Interestingly variable temperature studies (323 and 343 K) suggest that the structural integrity of **8** is maintained at higher temperatures, as indicated by the absence of any changes in the ^1^H NMR spectrum.

In the case of compound **9**, a single diffusion coefficient is observed at 5.05 × 10^–10^ m^2^ s^–1^ (Figure S14). This corresponds to a hydrodynamic radius of 6.74 Å, *cf.* 5.90 Å in the solid state, while using the ECC method a molecular weight of 1024 g mol^–1^ is determined, *cf.* a calculated value of 1096 g mol^–1^. This again suggests that the dimeric structure obtained in solution is retained in the solid state.

### Molecular Structures of Compounds **6–9**

**Figure 8:**
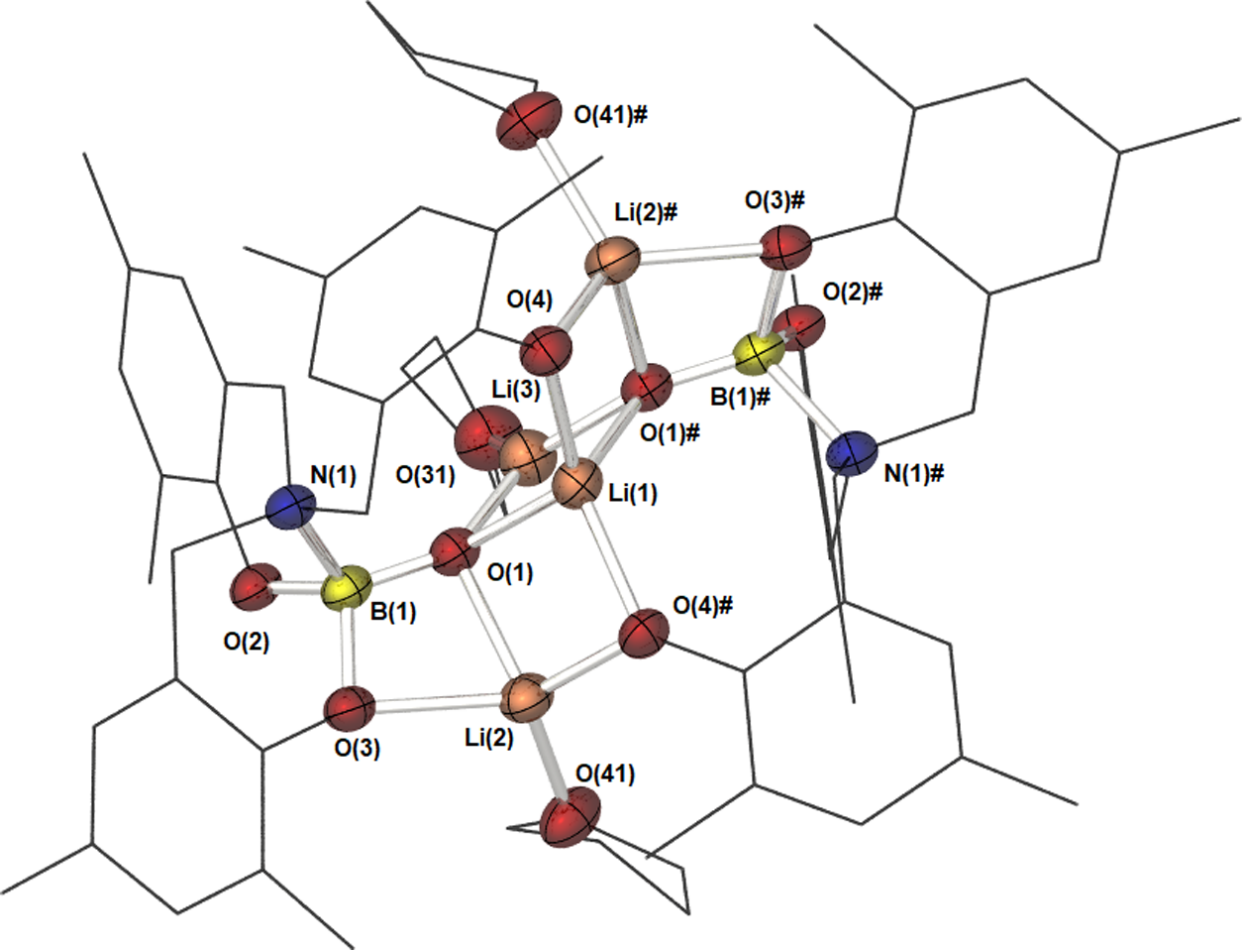
Molecular structure of compound **6**. Thermal ellipsoids are shown at 50% probability. Hydrogen atoms and THF found in the unit cell have been omitted and ligand framework shown as wires for clarity. Equivalent atoms are generated by the symmetry operator: # = 3/2 – *X*, *Y*, 1 – *Z*.

The lithium complex, **6**, crystallizes in the space group *I*2/*a* as a symmetric tetra-lithium dimer (Figure 8). Each unit cell contains one half of the dimer alongside THF as solvent of crystallization. The molecule contains two molecules of **3** and four lithium centers, which are found in three different environments. These form a twisted ladder comprising five planar four-membered rings (∑_∠_ = 360° in all cases), three {Li_2_O_2_}, and two {LiBO_2_}. The boryloxy oxygen (O1) displays a μ^3^ coordination mode, with each one coordinating to all three of the different lithium environments. Selected bond lengths and bond angles are shown in Table 4.

**Table 4:** Selected Bond Lengths and Angles from Compound **6**

bond lengths (Å)			
Li(1)–O(1)	1.962(3)	Li(3)–O(1)	1.845(3)
Li(1)–O(4)	1.951(2)	Li(3)–O(31)	1.912(6)
Li(2)–O(1)	1.952(4)	B(1)–O(1)	1.682(2)
Li(2)–O(3)	2.078(4)	B(1)–O(2)	1.360(3)
Li(2)–O(4)#	1.863(4)	B(1)–O(3)	1.479(2)
Li(2)–O(41)	1.962(4)	B(1)–N(1)	1.499(2)

bond angles (deg)			
O(1)–Li(1)–O(1)#	96.8(2)	O(3)–B(1)–N(1)	104.23(15)
O(1)–Li(1)–O(4)	120.95(8)	Li(1)–O(1)–Li(2)	83.44(13)
O(1)–Li(1)–O(4)#	93.66(6)	Li(1)–O(1)–Li(3)	78.90(17)
O(4)–Li(1)–O(4)#	128.4(3)	Li(1)–O(1)–B(1)	130.22(15)
O(1)–Li(2)–O(3)	71.60(12)	Li(2)–O(1)–Li(3)	119.70(13)
O(1)–Li(2)–O(4)	96.80(16)	Li(2)–O(1)–B(1)	92.99(15)
O(1)–Li(2)–O(41)	122.2(2)	Li(3)–O(1)–B(1)	140.39(16)
O(3)–Li(2)–O(4)#	129.68(19)	Li(2)–O(3)–B(1)	84.22(14)
O(1)–Li(3)–O(1)#	105.4(3)	Li(2)–O(3)–C(10)	148.01(16)
O(1)–Li(3)–O(31)	127.32(13)	B(1)–O(3)–C(10)	121.89(15)
O(1)–B(1)–O(2)	118.05(17)	Li(1)–O(4)–Li(2)#	86.10(14)
O(1)–B(1)–N(1)	111.29(15)	Li(1)–O(4)–C(19)	136.29(13)
O(2)–B(1)–N(1)	104.06(14)	Li(2)#–O(4)–C(19)	134.36(16)

Li(1) is a distorted tetrahedron (τ_4_′ = 0.76),^36^ coordinating to both boryloxy oxygen atoms, O(1), and both phenolic oxygens, O(4). Both bond lengths are longer than average, but commensurate with other μ^3^-boroxides^50^ and μ^2^-phenoxides.^51,52^ Li(2), which appears twice in the symmetry-generated dimeric structure, is also a distorted tetrahedron (τ_4_′ = 0.74), showing coordination to one μ^3^ boroxide center, O(1), one μ^2^ phenolic center, O(4) and a coordinated molecule of THF. The final interaction arises via a weaker dative interaction with O(3) that is otherwise coordinated to the boron center. The Li(2)–O(3) bond length is elongated as is the B(1)–O(3) bond length, a secondary effect of this dative interaction. The third lithium environment, Li(3), has a trigonal planar geometry (∑_∠_ = 360.0°), binding to the two, symmetry-generated boryloxy centers, O(1), and a molecule of THF. The Li–O(1) boryloxy bond is the shortest found within the molecule, being shorter than other μ^3^ boryloxy bonds but commensurate with other {B–O} bonds to three-coordinate lithium centers.^10,13^

The B–O(1) bond length is significantly elongated in comparison to both that observed in **3** and other {Li–O–B}-containing species reported elsewhere. As a result of this, the B–N bond length is significantly shortened, demonstrating a stronger dative interaction to the boron center, demonstrating the ability of these systems to act as electron-rich donor ligands. Despite these adjustments, only a small shift in the ^11^B NMR resonance is observed. The boron center retains a tetrahedral geometry, with no significant deviations (τ_4_′ = 0.91). The interaction between O(3) and Li(2) means the two phenolic oxygens are in significantly different environments. Not unexpectedly, the B–O(3) bond length is observed to lengthen in comparison to the B–O(2) bond.

**Figure 9:**
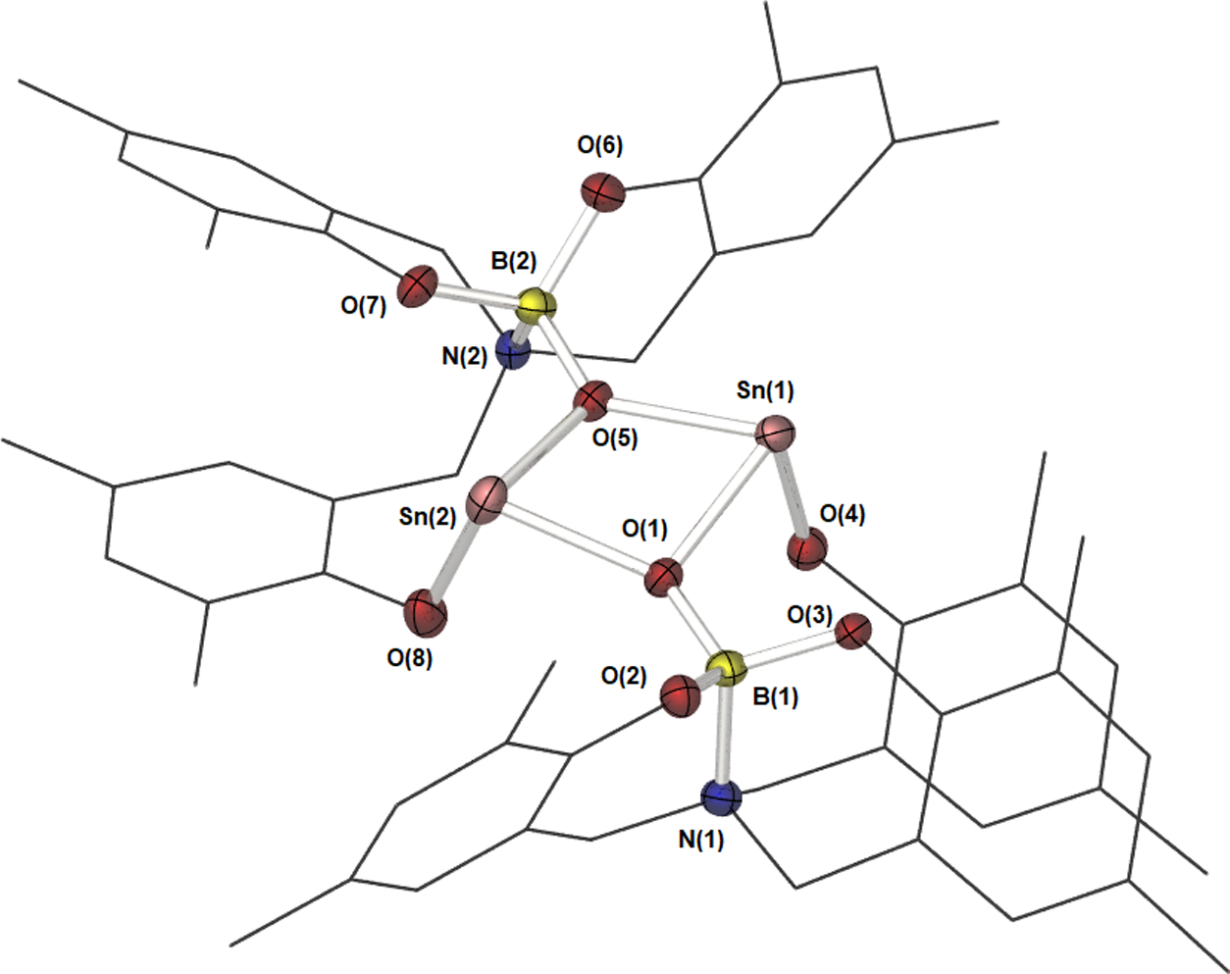
Molecular structure of compound **7**. Thermal ellipsoids are shown at 50% probability. Hydrogen atoms have been omitted, and ligand framework is shown as wires for clarity.

Compound **7**, which crystallizes in the triclinic space group *P*1̅ alongside three disordered THF molecules as solvent of crystallization, is a molecular dimer, comprising two Sn centers and 2 equiv of compound **3** (Figure 9). Selected bond lengths and bond angles are shown in Table 5. While crystallographically inequivalent, the differences are inconsequential, and both are equivalent in the solution state (as shown by NMR), suggesting that this is a crystal packing effect. The Sn center has a pseudo-trigonal pyramidal geometry, with the bond angles about the Sn center indicative of a constrained geometry. Puckering is observed in the {Sn_2_O_2_} core (∑_∠_ = 380°), resulting in a saddle-like geometry. The lariat hydroxybenzyl groups (O4/8) bind via a terminal μ^1^ coordination mode, while the boryloxy groups display a bridging μ^2^ coordination mode. There is only a slight difference observed in the bond lengths between the two Sn centers, suggestive of a strongly held dimer. The two previously reported Sn(II)–O–B systems (Figure 3; **F** and **I**) feature significantly shorter Sn–O and B–O bonds, albeit both display a μ^1^-O boryloxy binding mode (Sn–O = 2.015/2.041 Å, O–B = 1.353/1.317 Å, *cf.* Sn–O = 2.14 Å and B–O = 1.407 Å here).^25,30^ The longer B–O bond found in compound **7** is suggestive of a stronger interaction between the oxygen atom and the tin center, an observation supported by the highly shielded resonance observed in the ^119^Sn NMR spectrum of compound **7**. The Sn(1)–O(4) bond length is shorter than the corresponding boryloxy bond, indicative of the expected stronger interaction from the phenolic center; however, this bond is longer than that observed in similar dimeric tin phenoxide complexes.^38^

**Table 5:** Selected Bond Lengths and Angles from Compound **7**

bond lengths (Å)			
Sn(1)–O(1)	2.151(3)	Sn(2)–O(1)	2.126(3)
Sn(1)–O(4)	2.097(3)	Sn(2)–O(5)	2.150(3)
Sn(1)–O(5)	2.124(3)	Sn(2)–O(8)	2.085(3)
B(1)–O(1)	1.407(5)	B(2)–O(5)	1.416(5)
B(1)–O(2)	1.457(5)	B(2)–O(6)	1.461(5)
B(1)–O(3)	1.451(5)	B(2)–O(7)	1.455(5)
B(1)–N(2)	1.654(5)	B(2)–N(2)	1.641(5)
Sn(1)–Sn(2)	3.363(4)		

bond angles (deg)			
O(1)–Sn(1)–O(4)	86.36(10)	O(5)–Sn(2)–O(8)	89.50(11)
O(1)–Sn(1)–O(5)	71.87(10)	O(1)–Sn(2)–O(5)	71.86(10)
O(4)–Sn(1)–O(5)	92.51(10)	O(1)–Sn(2)–O(8)	92.09(11)
Sn(1)–O(1)–Sn(2)	103.69(11)	Sn(1)–O(5)–Sn(2)	103.80(11)
Sn(1)–O(1)–B(1)	115.0(2)	Sn(1)–O(5)–B(2)	133.8(2)
Sn(1)–O(5)–B(2)	136.4(2)	Sn(2)–O(5)–B(2)	117.9(2)
Sn(1)–O(4)–C(19)	115.5(2)	Sn(2)–O(8)–C(49)	113.9(3)
O(1)–B(1)–O(2)	115.8(3)	O(5)–B(2)–O(6)	114.9(3)
O(1)–B(1)–O(3)	108.0(3)	O(5)–B(2)–O(7)	107.2(3)
O(1)–B(1)–N(1)	109.5(3)	O(5)–B(2)–N(2)	109.4(3)
O(2)–B(1)–O(3)	109.2(3)	O(6)–B(2)–O(7)	110.1(3)
O(2)–B(1)–N(1)	106.0(3)	O(6)–B(2)–N(2)	107.0(3)

Bond lengths and angles around the boron center are similar to those found in compound **3**, indicating little change in the electron density of the boron center upon coordination. Such an observation is supported by NMR studies, with minimal change in the position of the ^11^B resonance of **7** (δ = 2.33 ppm in **3***cf.* δ = 2.63 ppm in **7**). Slight differences in bond angles about the B center are observed (τ_4_′ = 0.97).

**Figure 10:**
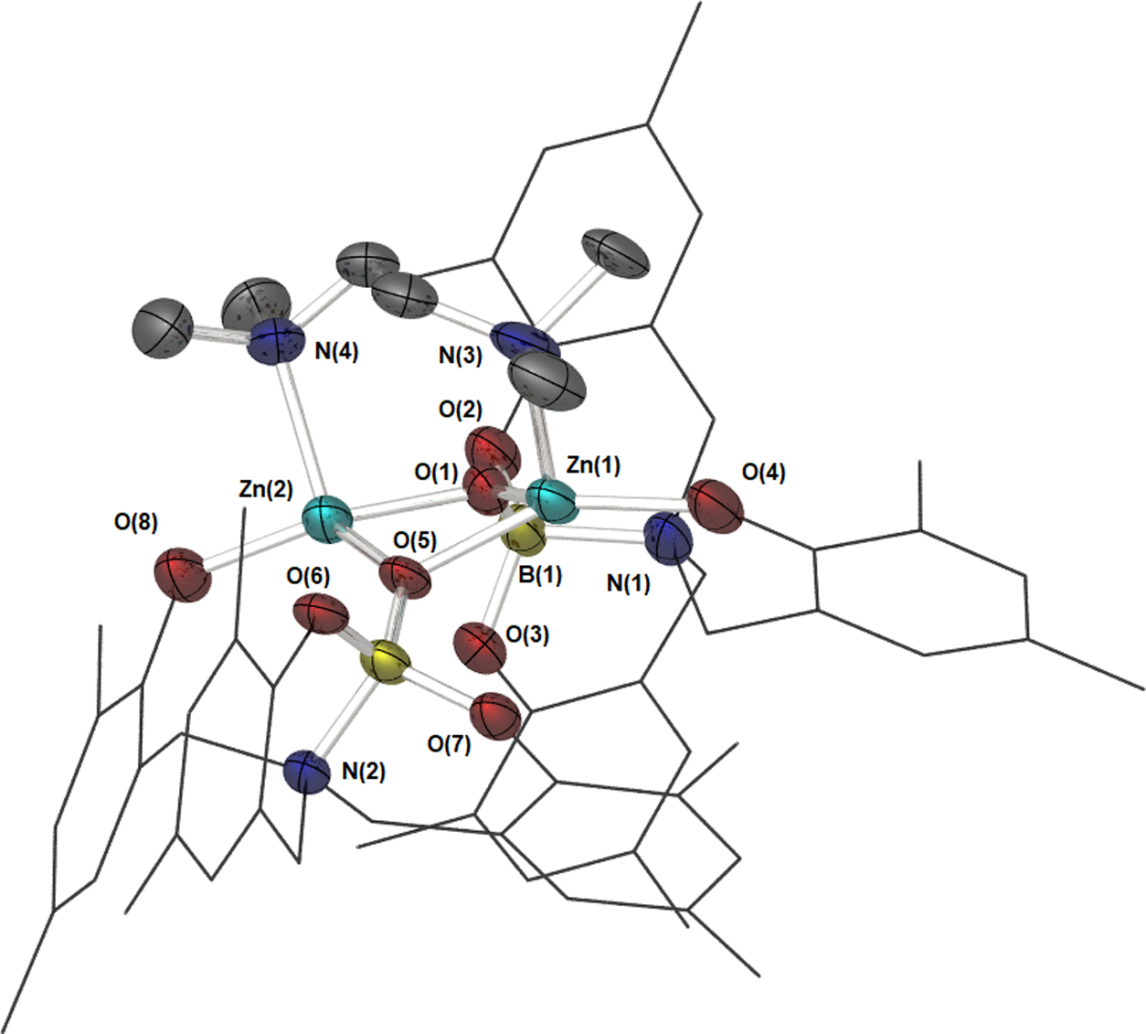
Molecular structure of compound **8** showing the relative ligand distribution about the {Zn_2_O_2_} core. Thermal ellipsoids are shown at 50% probability. Hydrogen atoms and benzene found in the unit cell have been omitted, and ligand framework is shown as wires for clarity.

Compound **8** crystallizes in the space group *P*21/*n* via slow evaporation of benzene. The unit cell contains two molecules of benzene as solvent of crystallization. The resulting molecular structure is a molecular dimer, in which the boryloxy ligand displays μ^2^-coordination through the boryloxy oxygen atom O(1/5), and μ^1^-coordination through the lariat phenoxide, O(4/8). The structure features 1 equiv of the donor base, which demonstrates a unique μ^2^κ^2^-binding mode, bridging the {Zn_2_O_2_} core (Figure 10). Selected bond lengths and bond angles are shown in Table 6.

**Table 6:** Selected Bond Lengths and Angles from Compound **8**

bond lengths (Å)			
Zn(1)–O(1)	1.976(4)	Zn(2)–O(1)	1.970(4)
Zn(1)–O(4)	1.897(4)	Zn(2)–O(5)	1.947(4)
Zn(1)–O(5)	1.954(3)	Zn(2)–O(8)	1.867(4)
Zn(1)–N(3)	2.118(6)	Zn(2)–N(4)	2.115(5)
B(1)–O(1)	1.396(9)	B(2)–O(5)	1.405(7)
B(1)–O(2)	1.457(7)	B(2)–O(6)	1.450(8)
B(1)–O(3)	1.458(7)	B(2)–O(7)	1.474(7)
B(1)–N(2)	1.662(8)	B(2)–N(2)	1.654(7)
Zn(1)–Zn(2)	2.8059(10)		

bond angles (deg)			
O(1)–Zn(1)–O(4)	125.35(19)	O(5)–Zn(2)–O(8)	123.61(16)
O(1)–Zn(1)–O(5)	85.97(15)	O(1)–Zn(2)–O(5)	138.9(2)
O(4)–Zn(1)–O(5)	140.38(16)	O(1)–Zn(2)–O(8)	86.34(16)
O(1)–Zn(1)–N(3)	111.41(19)	O(1)–Zn(2)–N(4)	96.76(18)
O(4)–Zn(1)–N(3)	93.2(2)	O(5)–Zn(2)–N(4)	112.6(2)
O(5)–Zn(1)–N(3)	96.71(19)	O(8)–Zn(2)–N(4)	96.0(2)
Zn(1)–O(1)–Zn(2)	90.63(17)	Zn(1)–O(5)–Zn(2)	92.01(15)
Zn(1)–O(1)–B(1)	133.4(4)	Zn(2)–O(5)–B(2)	138.4(3)
Zn(1)–O(5)–B(2)	128.0(3)	Zn(2)–O(1)–B(1)	122.4(3)
Zn(1)–O(4)–C(19)	137.5(5)	Zn(2)–O(8)–C(49)	131.6(4)
O(1)–B(1)–O(2)	115.1(5)	O(5)–B(2)–O(6)	111.8(5)
O(1)–B(1)–O(3)	112.8(5)	O(5)–B(2)–O(7)	112.9(5)
O(1)–B(1)–N(1)	108.6(5)	O(5)–B(2)–N(2)	110.2(5)
O(2)–B(1)–O(3)	108.2(5)	O(6)–B(2)–O(7)	109.4(5)
O(2)–B(1)–N(1)	105.3(5)	O(6)–B(2)–N(2)	105.1(4)

To the best of our knowledge, binding of a TMEDA ligand across the two metal centers of an {M_2_O_2_} core unprecedented with only three other examples of intramolecular μ^2^κ^2^-binding modes reported previously on Li and Mn complexes.^53−55^

The Zn center displays a highly distorted tetrahedral geometry (τ_4_′ = 0.71) and the {Zn_2_O_2_} core is highly puckered (∑_∠_ = 432°), likely the result of the μ^2^κ^2^-TMEDA coordination mode. The similar Zn–O(1) and Zn–O(5) bond lengths are indicative of a strongly held dimer, and are observed to be commensurate with those reported in similar borinic acid derivatives (Figure 3; **D**).^10,17^ The B–O bond is longer than in the same borinic acid derivatives, though this does not alter the geometry around the boron center, which remains a near-perfect tetrahedron (τ_4_′ = 0.94). This bond appears slightly shorter upon complexation to the Zn center in contrast to that found in **3**. The Zn(1)–O(4) bond length is slightly longer than previously reported μ^1^ zinc phenoxide systems.^56,57^ It is interesting to note that despite the ^1^H and ^13^C{^1^H} NMR spectra showing distinct resonances for all six aromatic rings, little difference is observed in the bond lengths and angles. Each half is crystallographically different, and while this would normally be attributed to solid-state packing effects, the observation that these slight spatial discrepancies are retained in the solution phase is a testament to the remarkable rigidity of these systems.

**Figure 11:**
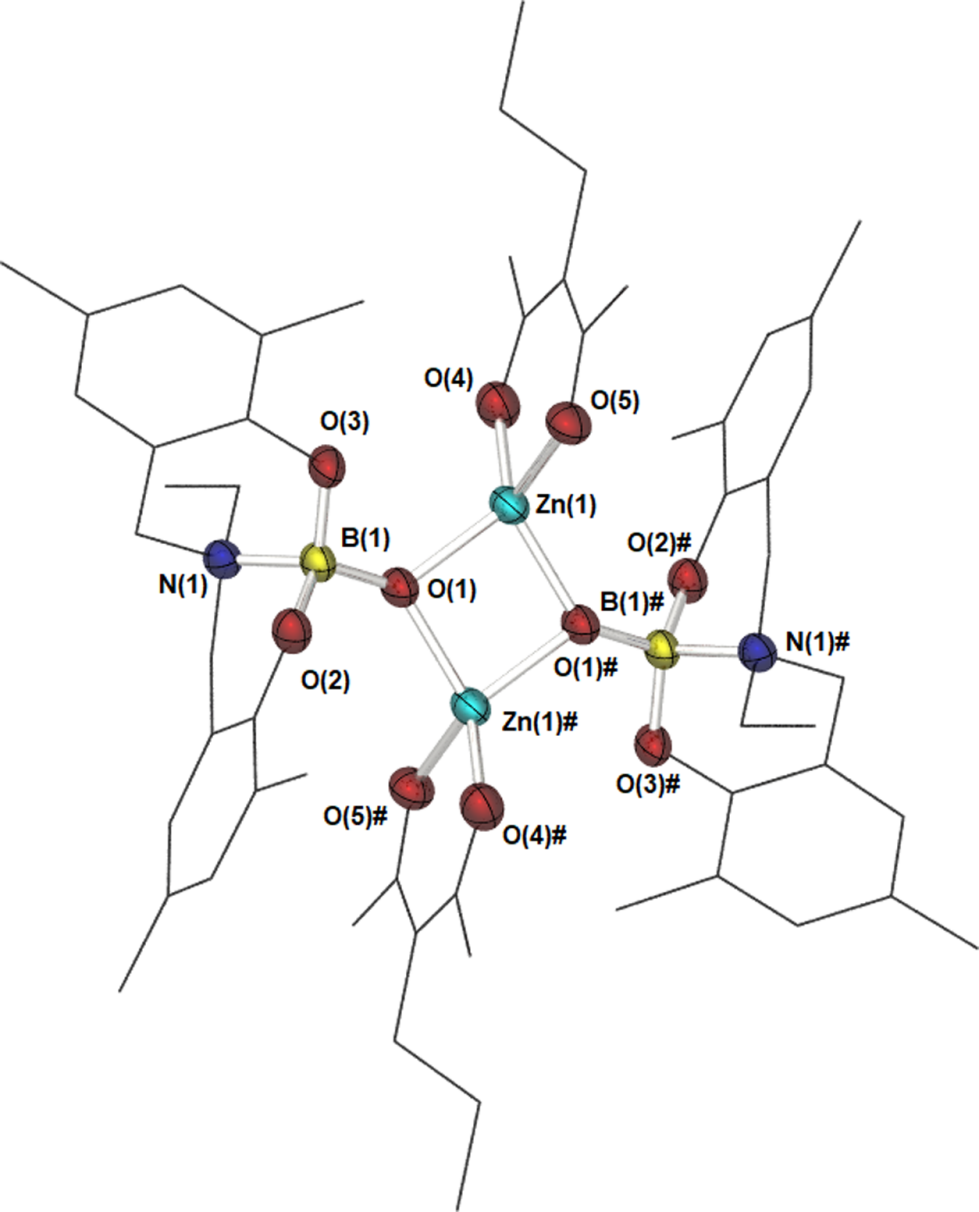
Molecular structure of compound **9**. The unit cell contains two monomeric halves, with equivalent atoms generated by the symmetry operators: # = −*X*, 1 – *Y*, −*Z* and $ = 1 – *X*, 1 – *Y*, 1 – *Z*. Thermal ellipsoids are shown at 50% probability. Hydrogen atoms, solvent, and the second unit present within the unit cell have been omitted, and the ligand framework is shown as wires for clarity.

Compound **9** crystallizes in the space group *P*21/*c*, alongside three molecules of DCM as solvent of crystallization. While the molecule exists as a dimer (Figure 11), the unit cell contains two halves of separate dimeric units (Figure S2) with the second half of each molecule generated via symmetry. While symmetrically inequivalent, the differences between the two molecules are inconsequential. Each dimer comprises a κ-O,O′-AcAc ligand, alongside a μ^2^-boryloxy center, resulting in a {Zn_2_O_2_} central core. Much like in **8**, the Zn center adopts a highly distorted tetrahedral geometry (τ_4_′ = 0.80), with constrained angles arising both within the AcAc ligand and between the two boryloxy ligands. Selected bond lengths and bond angles are shown in Table 7.

**Table 7:** Selected Bond Lengths and Angles from Compound **9**

bond lengths (Å)			
Zn(1)–Zn(1)#	2.8528(6)	Zn(2)–Zn(2)$	2.8362(6)
Zn(1)–O(1)	1.9524(17)	Zn(2)–O(11)	1.9314(16)
Zn(1)–O(1)#	1.9393(16)	Zn(2)–O(11)$	1.9499(17)
Zn(1)–O(4)	1.9234(19)	Zn(2)–O(14)	1.9318(18)
Zn(1)–O(5)	1.9337(17)	Zn(2)–O(15)	1.9196(19)
B(1)–O(1)	1.390(3)	B(2)–O(11)	1.400(3)
B(1)–O(2)	1.472(3)	B(2)–O(12)	1.452(3)
B(1)–O(3)	1.453(3)	B(2)–O(13)	1.462(3)
B(1)–N(1)	1.661(3)	B(2)–N(2)	1.669(3)

bond angles (deg)			
O(1)–Zn(1)–O(1)#	85.71(7)	O(11)–Zn(2)–O(11)$	86.10(7)
O(1)–Zn(1)–O(4)	123.38(8)	O(11)–Zn(2)–O(14)	124.06(8)
O(1)–Zn(1)–O(5)	112.96(8)	O(11)–Zn(2)–O(15)	116.58(8)
O(4)–Zn(1)–O(5)	94.78(8)	O(14)–Zn(2)–O(15)	95.58(9)
Zn(1)–O(1)–Zn(1)#	94.29(7)	Zn(2)–O(11)–Zn(2)$	93.90(7)
Zn(1)–O(1)–B(1)	134.80(16)	Zn(2)–O(11)–B(2)	127.54(16)
Zn(1)#–O(1)–B(1)	126.12(15)	Zn(2)$-O(11)–B(2)	134.72(16)
Zn(1)–O(4)–C(22)	124.63(19)	Zn(2)–O(14)–C(52)	123.6(2)
Zn(1)–O(5)–C(24)	123.71(19)	Zn(2)–O(15)–C(54)	123.4(2)
O(1)–B(1)–O(2)	114.4(2)	O(11)–B(2)–O(12)	111.8(2)
O(1)–B(1)–O(3)	111.46(19)	O(11)–B(2)–O(13)	113.9(2)
O(1)–B(1)–N(1)	110.0(2)	O(11)–B(2)–N(2)	109.3(2)
O(2)–B(1)–O(3)	109.3(2)	O(12)–B(2)–O(13)	109.6(2)
O(2)–B(1)–N(1)	104.75(18)	O(12)–B(2)–N(2)	105.45(18)

The Zn–O bond lengths to the {AcAc} ligand are commensurate with those observed in [Zn(AcAc)_2_].^58^ The associated bond angles (both Zn–O–C and O–Zn–O) show slight deviations for those observed in [Zn(AcAc)_2_], with the ligand bite angle constrained (94.78° here *cf.* 97.26° in [Zn(AcAc)_2_]).

The μ^2^-coordination mode of the boryloxy ligand is similar to that observed in **8**, with the two Zn–O bonds of similar lengths, suggestive of a strongly held dimer. The angles within the {Zn_2_O_2_} core (∑_∠_ = 397°) indicate puckering of the four-coordinate ring, albeit significantly less than that observed in **8**. The Zn–O bond length here is shorter than those found in **8** and other μ^2^ borinic acid derivatives (Figure 3; **D**),^10,17^ with the corresponding B–O bond length showing the opposite trend. Such an observation is consistent with the presence of increased electron density on the boron atom, a feature confirmed by density functional theory (DFT) calculations (vide infra), a result of the intramolecular B ← N interaction, which in turn, results in a stronger O–Zn interaction. The B ← N bond length is analogous to that observed in **5**, while the B–O bond length is slightly shorter here. The boron center remains a near-perfect tetrahedron (τ_4_′ = 0.96), and aside from the B–O(1) bond highlighted, bond lengths about the boron center show no significant change from those found in compound **5**. This is borne out in the ^11^B NMR, which shows only a slight change upon complexation (δ = 2.40 ppm in **4**, *cf.* δ = 2.82 ppm in **9**).

## DFT Studies

**Figure 12:**
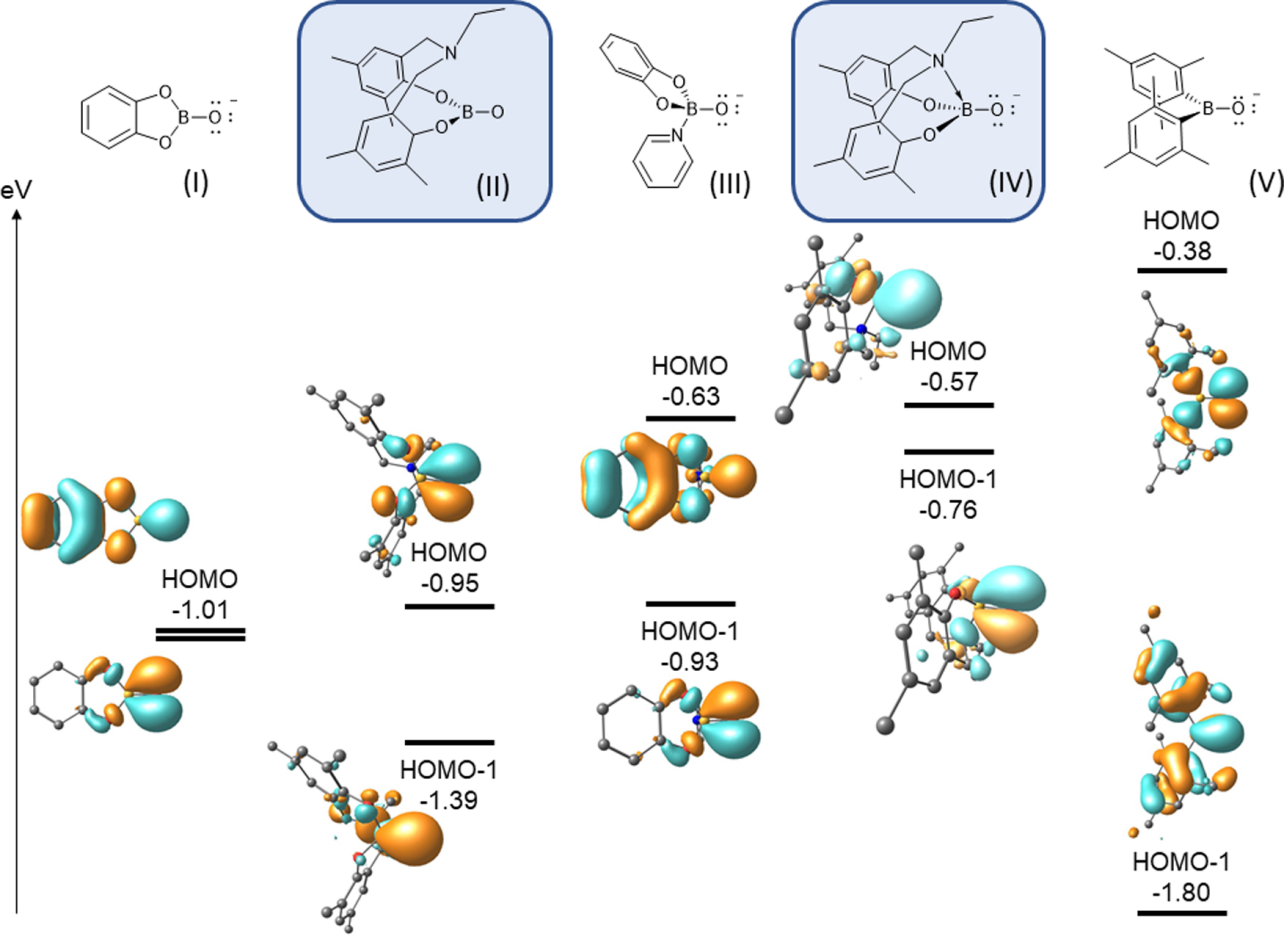
Two highest occupied molecular orbitals (frontier orbitals) and their relative energies of boryloxo anions (**I**–**IV**), and the boroxide anion (**V**) (BP86/6-31++G**//BP86/6-31G**, NBO7).

As part of our study, density functional theory (DFT) calculations were performed to ascertain the relative donor capabilities of the boryloxy ligands described here, compared to a range of selected oxygen donor ligands (I–V: Figure 12). These include the catechol-based anions **I** and **III**, alongside the bis-mesityl boroxide anion, **V**. The amino-bis-phenoxy-boryloxy anions, **II** and **IV**, differ only by virtue of the interaction between the boron center and the bridge head nitrogen of the bis-phenol amine moiety: For anion **II**, the amino functionality, {EtN}, is noncoordinating and as such the {O_2_BO} unit is trigonal planar. In contrast, boryloxy anion, **IV**, exhibits a pseudo-tetrahedral coordination geometry about the boron atom by virtue of coordination of the amino functionality to the boron atom.

Employing the BP86 functional and 6-31++G** basis set (see the Supporting Information) on the “free” anionic pro-ligands, DFT calculations show the isolobal nature of these systems: the highest occupied molecular orbital (HOMO) and HOMO – 1 in each case are the orthogonal in- and out-of-plane π-donor orbitals, although their relative ordering varies according to the π-acceptor capabilities of the O- bound group. In general, the energies of the HOMO/HOMO – 1 orbitals rise in order from **I–V**, Table 8. In all cases, the σ-orbitals associated with each of the ligands are lower in energy than the frontier π-orbitals, while the LUMO is based on atoms away from the core unit, except in the case of anion **III**, where the LUMO is based across the pyridine-{BO_3_} part of the anion.

**Table 8:** Summary of DFT Results (BP86/6-31++G**//BP86/6-31G**) and NPA - Charges (NBO7) of Selected Atoms in “Free” the Anions **I–V** (Figure 12)

	MO energies (eV)				natural population analysis charges (C)		
compound	LUMO	HOMO	HOMO – 1	HOMO–LUMO gap	*q*(O)	*q*(B)	Δ*q* = *q*(B) – *q*(O)
**I**	2.20	–1.01	–1.01	3.21	–0.96	1.08	2.06
**II**	1.71	–0.95	–1.39	2.67	–1.02	1.16	2.18
**III**	0.65	–0.63	–0.93	1.28	–0.92	1.08	2.00
**IV**	1.69	–0.57	–0.76	2.26	–1.07	1.15	2.22
**V**	1.85	–0.38	–1.80	2.23	–0.94	0.83	1.77

Interestingly in the case of the amino-bis phenol-supported boryloxide ligands **II** and **IV**, the coordination of the amino functionality to the boron center in **IV** has a significant effect on the energies of the HOMO and HOMO – 1 orbitals raising the energies of both orbitals.

While DFT suggests that the HOMO of the bis-mesityl anion **V** is higher still than that of the anion **IV**, an inspection of the NPA (Natural Population Analysis) charges (*q*) for the boron and oxygen atoms in each anion (Table 8) also depicts significant variance in the electronic structures of these anions. In each case, the oxygen atom bears a partial negative charge, with a positive charge of roughly equal magnitude located on the boron atom. This relative charge distribution results in polar B–O bonds in all of the anions, with anion **IV** showing the highest degree of polarization (Δ*q* = 2.22), consistent with our initial hypothesis that the donation of electron density into the formally vacant p-orbital on boron, from an appended N-donor group would render the boryloxy ligands stronger π-donor ligands.

## Conclusions

In conclusion, we present here our preliminary investigations into a new class of amine-stabilized, electron-rich metal boryloxy complexes, with selected examples from the s-, p-, and d-block elements, specifically Li (**6**), Sn(II) (**7**), and Zn (**8** and **9**).

Given the ubiquity of metal alkoxide chemistry and the ability of metal boroxide and boryloxy species to afford species with different electronic and steric properties, the relative rarity of these systems is perhaps surprising. Formed by the reaction of aminophenol ligands with boronic acid, the pro-ligand systems, **3** and **4**, represent a new class of ligands, specifically amino-tris-phenoxy-boryloxy ligands and amino-*bis*-phenoxy-boryloxy as shown in Scheme 1, the electronic and steric tunability of which is moderated by the availability of amino-phenol and amino-alcohol scaffolds.

Complexes of the amino-tris-phenoxy-boryloxy (Li, **6**, Sn, **7** and Zn, **8**) and amino-bis-phenoxy-boryloxy ligands (Zn, **9**) have been synthesized and characterized in both the solution and solid state. In all cases, bridging binding modes are observed (μ^3^ for Li, μ^2^ for Zn, Sn), consistent with the presence of an electron-rich {O–B} unit, which is supported by DFT calculations. We attribute this to the presence of electron donation from the amino group into the vacant p-orbital of the B atom, thereby increasing the electron density of the boryloxy group.

Employment of these ligands has enabled the successful stabilization of the first examples of metal complexes supported by phenoxy-*N*-boryloxy (**A**) and N-boryloxy ligands. While literature examples of boryloxy systems containing ligands of the form {(RO)_2_BO} are known, these systems are limited to the pinacol and catechol systems. Species created around other O-based scaffolds are conspicuously rare, with N-stabilized systems even rarer still. We foresee that this class of ligand, with its strong donor capacity and large steric profile, will provide an entry point to access a wide range of other oxy-stabilized metal species.

## Experimental Section

Complexes **1**–**5** were synthesized under ambient conditions using reagent-grade solvents that had not been subject to further purification. Complexes **6**–**9** were treated as air- and moisture-sensitive. All manipulations of air- and moisture-sensitive compounds were carried out under an atmosphere of nitrogen or argon using standard Schlenk-line or glovebox techniques. Solvents were dried according to standard methods and collected by distillation. All reagents were purchased from commercial sources and used without further purification. Ligand **L1**, *^n^*Pr-AcAc, [Sn{N(SiMe_3_)_2_}_2_], and [Zn{N(SiMe_3_)_2_}_2_] were prepared according to literature procedures.^33,59−61^

^1^H, ^11^B, ^13^C, and ^119^Sn NMR spectra were recorded on Bruker Avance 400 and 500 MHz FT-NMR spectrometers, in saturated solutions at 298 K. Chemical shifts are expressed in ppm with respect to Me_4_Si (^1^H and ^13^C), LiCl (^7^Li), BF_3_**·**OEt_2_ (^11^B), or Me_4_Sn (^119^Sn). DOSY experiments were carried out on a Bruker 500 MHz spectrometer at concentrations of 20 mM, using a standard double attenuated echo sequence with longitudinal eddy current delay. Experiments were typically carried out with a gradient strength ranging from 10 to 90% using smoothed square gradients, and with Δ and δ set to 100 and 1.2 ms, respectively. Data were processed using Bruker Dynamics Center. Elemental analysis was conducted by Exeter Analytical using an Exeter Analytical CE440 Elemental Analyzer. All samples were run in duplicate.

### *N*,*N*-Bis(3,5-dimethyl-2-hydroxybenzyl)ethylamine—**L2**

2,4-Dimethylphenol (12.2 g, 100.0 mmol) was added to a mixture of formaldehyde solution (8.12 mL of a 37% aq. solution, 50.0 mmol) and ethylamine (3.22 g of a 70% aq. solution, 50.0 mmol) at 0 °C. The reaction mixture was heated to reflux for 48 h. The white solid formed upon cooling was dissolved in DCM (100 mL) and washed with water (50 mL). The aqueous layer was subsequently washed with two further portions of DCM (2 × 30 mL). The combined organic layers were dried with Na_2_SO_4_ and concentrated under vacuum. The resulting solid was washed with cold methanol to give 9.56 g (61%) of white powder. Details of ^1^H and ^13^C{^1^H} NMR spectra are given in the Supporting Information. HRMS (ESI+): calculated for M^+^ C_20_H_27_NO_2_, 313.2042; found 313.2046.

### Aminotrisphenolatephenylborate (**1**)

A solution of phenylboronic acid (0.244 g, 2.0 mmol) and **L2** (0.839 g, 2.0 mmol) was combined in THF (25 mL) and stirred under ambient conditions for 16 h. Na_2_SO_4_ was added for the final hour of stirring. The reaction was filtered and concentrated under vacuum, giving a white residue. Recrystallization from chloroform at −28 °C gave 0.390 g (39%) of pale-yellow crystals. Details of ^1^H, ^11^B, and ^13^C{^1^H} NMR spectra are given in the Supporting Information. HRMS (ESI+): calculated for M^+^ C_33_H_36_BNO_3_, 504.2825; found, 504.2825. Elemental Analysis: Found (Calculated) C: 74.16 (74.60) H: 6.73 (6.83) N: 2.70 (2.62) −0.25 equiv of CHCl_3_ present as per asymmetric unit cell.

### Aminobisphenolatephenylborate (**2**)

A solution of phenylboronic acid (0.244 g, 2.0 mmol) and **L1** (0.627 g, 2.0 mmol) was combined in THF (25 mL) and stirred under ambient conditions for 16 h. The reaction was concentrated under vacuum, and the resulting white residue was redissolved in a minimum of DCM. Colorless crystals (0.421 g, 53%) were grown at room temperature via layering of hexane. Details of ^1^H, ^11^B, and ^13^C{^1^H} NMR spectra are given in the Supporting Information. HRMS (ESI+): calculated for M^+^ C_26_H_30_BNO_2_, 398.2406; found, 398.2410. Elemental Analysis: Found (Calculated) C: 77.85 (78.20) H: 7.68 (7.57) N: 3.55 (3.51)

### Aminotrisphenolborate (**3**)

A solution of B(OH)_3_ (3.09 g, 50.0) and **L2** (20.98 g, 50.0 mmol) was combined in THF (350 mL) and stirred under ambient conditions for 16 h. The reaction was concentrated under vacuum to give a white residue that was recrystallized from boiling toluene to give 17.21 g (77%) of the product as colorless crystals. Details of ^1^H, ^11^B, and ^13^C{^1^H} NMR spectra are given in the Supporting Information. HRMS (ESI+): calculated for M^+^ C_27_H_32_BNO_4_, 444.2461; found, 444.2461. Elemental Analysis: Found (calculated) C: 73.00 (72.82) H: 7.24 (7.24) N: 3.13 (3.14)

### Aminobisphenolborate (**4**)

A solution of B(OH)_3_ (0.889 g, 14.4 mmol) and **L1** (4.51 g, 14.4 mmol) was combined in THF (150 mL) and stirred under ambient conditions for 16 h. Na_2_SO_4_ (0.50 g) was added for the last hour of the reaction. The reaction was filtered and concentrated under vacuum to give a white residue. This residue was washed with DCM (3 × 20 mL) to give a white powder. The resulting white powder was suspended in hexane (20 mL) and isolated via filtration, before being dried under high vacuum to give 3.29 g (68%) of the product as a white powder. Details of ^1^H, ^11^B, and ^13^C{^1^H} NMR spectra are given in the Supporting Information. HRMS (ESI+): calculated for M^+^ C_20_H_26_BNO_3_, 338.2042; found, 338.2045. Elemental Analysis: Found (Calculated) C: 71.24 (70.81) H: 7.88 (7.73) N: 3.52 (4.13)

### [Li_2_{Aminotrisphenolboryl}]_2_ (**6**)

A solution of **3** (0.891 g, 2.0 mmol) in THF (10 mL) was added to a solution of Li[N(SiMe_3_)_2_] (0.669 g, 4.0 mmol) in THF (10 mL). The reaction was stirred for 30 min, before being concentrated under vacuum to approximately 10 mL. Colorless crystals (0.770 g, 68%) were obtained upon standing at −28 °C. Details of ^1^H, ^7^Li, ^11^B, and ^13^C{^1^H} NMR spectra are given in the Supporting Information. Elemental Analysis: Found (calculated) C: 69.61 (70.10) H: 7.81 (7.49) N: 2.96 (2.48).

### [Sn{Aminotrisphenolboryl}]_2_ (**7**)

A solution of **3** (0.445 g, 1.0 mmol) in THF (10 mL) was added to a solution of Sn[N(SiMe_3_)_2_]_2_ (0.445 g, 1.0 mmol) in THF (10 mL). The reaction turned colorless and was stirred for 30 min. The reaction was filtered and concentrated under vacuum to approximately 10 mL. The resulting solution was left at 4 °C overnight, during which time the product crystallized, to give 0.232 g (41%) of the product as white crystals. Details of ^1^H, ^11^B, ^13^C{^1^H}, and ^119^Sn NMR spectra are given in the Supporting Information. Elemental Analysis: Found (Calculated) C: 60.30 (60.05) H: 6.24 (5.76) N: 2.47 (2.50).

### [{Zn(Aminotrisphenolboryl)}_2_{TMEDA}] (**8**)

A solution of **3** (0.445 g, 1.0 mmol) in THF (10 mL) was added to a solution of Zn{N(SiMe_3_)_2_}_2_ (0.386 g, 1.0 mmol) in THF (15 mL). The reaction was stirred for 15 min, after which *N*,*N*,*N*′,*N*′-tetramethylethylenediamine (0.116 g, 0.149 mL, 1.0 mmol) was added, and the reaction was left to stir at room temperature for a further 1 h. The solvent was removed under vacuum before the resulting white solid was recrystallized from toluene to give 0.262 g (46%) of white crystals of the product. Crystals suitable for X-ray diffraction were grown via the slow evaporation of C_6_D_6_. Details of ^1^H, ^11^B, and ^13^C{^1^H} NMR spectra are given in the Supporting Information. Elemental Analysis: Found (Calculated) C: 64.68 (65.09) H: 7.07 (7.28) N: 4.42 (4.60)—one molecule of C_6_D_6_ present as per asymmetric unit cell.

### [Zn{Aminobisphenolboryl}{*^n^*PrAcAc}]_2_ (**9**)

A solution of **4** (0.339 g, 1.0 mmol) in THF (15 mL) was added to a solution of Zn[N(SiMe_3_)_2_]_2_ (0.386 g, 1.0 mmol) in THF (15 mL). The reaction turned yellow and was stirred for 15 min. The solvent was removed, and the residue dried under vacuum before being redissolved in THF (15 mL). 3-*n*-Propyl-2,4-pentanedionate (0.142 g, 1.0 mmol) was subsequently added, and the reaction was stirred for a further 1 h. The solvent was removed under vacuum to give a yellow residue that was subsequently recrystallized from a 2:1 mixture of hexane and DCM at 4 °C, to give 0.150 g (14%) of the product as yellow crystals. Details of ^1^H, ^11^B, and ^13^C{^1^H} NMR spectra are given in the Supporting Information. Elemental Analysis: Found (calculated) C: 61.31 (61.73) H: 7.29 (7.03) N: 2.91 (2.57).

### Single-Crystal X-ray Diffraction

Experimental details relating to the single-crystal X-ray crystallographic studies for compounds **1–3** and **5–9** are summarized in Tables S1 and S2 (see the Supporting Information). All crystallographic data were collected at 150(2) K either on an Agilent Xcalibur or Agilent SuperNova, Dual, EosS2 diffractometer using radiation Cu Kα (λ = 1.54184 Å) or Mo Kα (λ = 0.71073 Å). All structures were solved by direct methods followed by full-matrix least-squares refinement on F^2^ using the WINGX-2014 suite of programs^62^ or OLEX2.^63^ All hydrogen atoms were included in idealized positions and refined using the riding model. Crystals were isolated from a round-bottom flask under ambient conditions or an argon-filled Schlenk flask and immersed in oil before being mounted onto the diffractometer. CSD 2194565-2194572 contains the supplementary crystallographic data for this paper. These data can be obtained free of charge at www.ccdc.cam.ac.uk/conts/retrieving.html or from the Cambridge Crystallographic Data Centre, 12, Union Road, Cambridge CB2 1EZ, UK; fax: +44-1223/336-033; E-mail: deposit@ccdc.cam.ac.uk.

### Computational Methodology

DFT calculations were run with Gaussian 16 (C.01),^64^ the 6-31G** basis set was used for all atoms^65,66^ with initial BP86 optimizations^67,68^ performed using the “grid = ultrafine” option, and all stationary points being fully characterized via analytical frequency calculations as minima (all positive eigenvalues). Natural Bonding Orbital (NBO7)^69^ analyses were performed on these BP86/6-31G** optimized geometries at the BP86/6-311++G** level, within Gaussian 16 (C.01).

## Supporting Information Available

^1^H, ^13^C{^1^H}, and DOSY NMR spectra and assignments of compounds **6**–**9**, and additional crystallographic data, including the structure of compound **5** and crystal and structure refinement for compounds **1–3** and **5**–**9** (PDF)
