# Supplementary material for: Lithium, Tin(II), and Zinc Amino-Boryloxy Complexes: Synthesis and Characterization
Source: Inorg Chem. 2023 Jan 28;62(6):2576–91. doi: 10.1021/acs.inorgchem.2c03108 (PMC9930121; doi:10.1021/acs.inorgchem.2c03108)
Supplement: Supplementary file 1 — ic2c03108_si_001.pdf [file ic2c03108_si_001.pdf]

Supporting Information

Lithium, Tin(II) and Zinc Amino-Boryloxy Complexes: Synthesis and Characterisation

Andrew J. Straiton,[a] Claire L. McMullin,[a] Gabriele Kociok- Köhn,[b] Catherine L. Lyall,[b] James D. Parish[c] and Andrew L. Johnson.[a]*

Contact: a.l.johnson@bath.ac.uk

[a] Department of Chemistry, University of Bath, Claverton Down, UK, BA2 7AY
[b] Material and Chemical Characterisation Facility, University of Bath, Claverton Down, UK, BA2 7AY
[c] Infineum UK Ltd., Milton Hill Business and Technology Centre, Abingdon, Oxfordshire, OX13 6BB

Table of Contents

NMR Assignments of L2 and Compounds 1-4 3
N,N-bis(3,5-dimethyl-2-hydroxybenzyl)ethylamine – L2 3
Aminotrisphenolatephenylborate (1) 3
Aminobisphenolatephenylborate (2) 3
Aminotrisphenolborate (3) 4
Aminobisphenolborate (4) 4
NMR Assignments of Compounds 6-9 5
Compound 6 5
Compound 7 6
Compound 8 6
Table S1: Complete ^{1}H (500 MHz) and $^{13}C\{^{1}H\}$ (125.7 MHz) NMR Assignment of Compound **8** in C_6D_6. 7

Compound 9 8
Supplementary Crystallographic Data 9
Table S2: Crystal and structure refinement data for compounds **1**, **2**, **3** and **5**. 9
Figure S1: The molecular structure of compound **5**. Thermal ellipsoids are shown at 50 % probability. Hydrogen atoms are omitted for clarity. 10
Table S3: Selected bond lengths and angles from compound **5**. 10
Figure S2: The asymmetric unit cell of compound **9**. This comprises two halves of the dimeric molecular structure. Equivalent atoms that afford the full dimeric structure are generated by the symmetry operators: # = -X, 1-Y, -Z and $ = 1-X, 1-Y, 1-Z. Thermal ellipsoids are shown at 50 % probability. Hydrogen atoms and solvent present within the unit cell have been omitted for clarity. 10
Table S4: Crystal and structure refinement data for compounds **6**, **7**, **8**, and **9**. 11
^{1}H, ^{13}C{^{1}H} and ^{1}H-DOSY NMR Spectra of Compounds 6-9 12
Figure S3: ^{1}H NMR spectrum of compound **6** in d_8-THF. 12
Figure S4: ^{13}C{^{1}H} NMR spectrum of compound **6** in d_8-THF. 12
Figure S5: ^{1}H-DOSY NMR spectrum of compound **6** in d_8-THF. 13
Figure S7: ^{13}C{^{1}H} NMR spectrum of compound **7** in C_6D_6. 14
Figure S8: ^{1}H-DOSY NMR spectrum of compound **7** in C_6D_6. 14
Figure S9: ^{1}H NMR spectrum of compound **8** in C_6D_6. 15
Figure S11: ^{1}H-DOSY NMR Spectrum of Compound **8** in C_6D_6. 16
Figure S12: ^{1}H NMR spectrum of compound **9** in C_6D_6. 16
Figure S13: ^{13}C{^{1}H} NMR spectrum of compound **9** in C_6D_6. 17
Figure S14: ^{1}H-DOSY NMR Spectrum of Compound **9** in C_6D_6. 17
DFT Studies 18
Cartesian Coordinates and Computed Energies (in Hartress) 18

NMR Assignments of L2 and Compounds 1-4

N,N-bis(3,5-dimethyl-2-hydroxybenzyl)ethylamine – L2

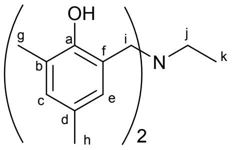

^{1}H NMR (500 MHz, C_6D_6) δ_H: 0.82 (3H, t, *J* = 7 Hz, CH_3-k), 2.16 (12H, s, ***Me***-h), 2.19 (6H, s, ***Me***-g), 2.39 (2H, q, *J* = 7 Hz, CH_2-j), 3.47 (4H, s, CH_2-i), 6.67 (2H, s, Ar***H***-e), 6.77 (2H, s, Ar***H***-c).

^{13}C{^{1}H} NMR (125.7 MHz, C_6D_6) δ_C: 10.7 (CH_3-k), 16.0 (***Me***-g), 20.6 (***Me***-h), 46.6 (CH_2-j), 55.7 (CH_2-i), 122.1 (***Ar***-f), 124.5 (***Ar***Me-b), 128.2 (***Ar***Me-d), 128.8 (***Ar***H-e), 131.4 (***Ar***H-c), 152.8 (***Ar***OH-a).

Aminotrisphenolatephenylborate (1)

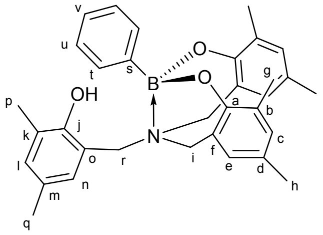

^{1}H NMR (500 MHz, CD_2Cl_2) δ_H: 2.18 (3H, s, ***Me***-q), 2.19 (3H, s, ***Me***-p), 2.24 (6H, s, ***Me***-h), 2.26 (6H, s, ***Me***-g), 3.91 (2H, d, *J* = 15 Hz, CH_2-i), 3.99 (2H, d, *J* = 15 Hz, CH_2-i), 4.00 (2H, s, CH_2-r), 6.56 (2H, s, Ar***H***-e), 6.57 (1H, s, Ar***H***-n), 6.97 (2H, s, Ar***H***-c), 6.98 (1H, s, Ar***H***-l), 7.22-7.23 (3H, m, BAr***H***-t,u), 7.61 (2H, dd, *J* = 2,8 Hz, Ar***H***-v).

^{11}B NMR (160.4 MHz, CD_2Cl_2) δ_B: 4.29.

^{13}C{^{1}H} NMR (125.7 MHz, CD_2Cl_2) δ_C: 16.1 (***Me***-p), 16.4 (***Me***-g), 20.4 (***Me***-q), 20.6 (***Me***-h), 51.7 (CH_2-r), 53.6 (CH_2-i), 115.7 (***Ar***CH_2-o), 116.6 (***Ar***CH_2-f), 123.9 (***Ar***Me-k), 125.5 (***Ar***H-e), 127.6 (***Ar***H-d/t), 128.0 (***Ar***H-u), 128.2 (***Ar***Me-d), 130.1 (***Ar***Me-m), 131.5 (***Ar***H-c), 133.1 (***Ar***H-l), 133.2 (***Ar***H-n), 133.7 (***Ar***H-v), 114.3 (***Ar***B-s), 150.2 (***Ar***OB-a), 152.0 (***Ar***OH-j).

Aminobisphenolatephenylborate (2)

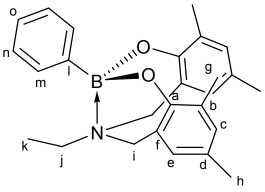

^{1}H NMR (500 MHz, CD_2Cl_2) δ_H: 1.05 (3H, t, *J* = 7 Hz, CH_3-k), 2.25 (12H, s, ***Me***-g,h), 2.90 (2H, q, *J* = 7 Hz, CH_2-j), 3.81 (2H, d, *J* =15 Hz, C***H***H-i[1]), 4.12 (2H, d, *J* =15 Hz, CH***H***-i[2]), 6.62 (2H, s, Ar***H***-e), 6.96 (2H, s, Ar***H***-c), 7.22 (3H, m, B***Ar***-n,o), 7.51 (2H, d, *J* = 7 Hz, B***Ar***-m).

^{11}B NMR (160.4 MHz, CD_2Cl_2) δ_B: 4.10.

^{13}C{^{1}H} NMR (125.7 MHz, CD_2Cl_2) δ_C: 6.2 (***CH₃***-k), 16.3 (***Me***-g), 20.5 (***Me***-h), 48.2 (N***CH₂***-j), 53.0 (***CH₂***-i), 115.9 (***Ar***-f), 125.3 (***Ar***H-e), 127.4 (B***Ar***), 127.5 (B***Ar***), 127.9 (***Ar***Me), 128.1 (***Ar***Me), 131.6 (***Ar***H-c), 133.5 (B***Ar***-m), 143.8 (B***Ar***-l), 150.1 (***Ar***OB-a).

Aminotrisphenolborate (3)

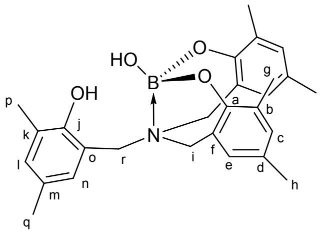

^{1}H NMR (500 MHz, CD_2Cl_2) δ_H: 2.15 (9H, s, ***Me***-g&q), 2.18 (6H, s, ***Me***-h), 2.21 (3H, s, ***Me***-p), 3.91 (2H, d, *J* = 15 Hz, N***CH₂***-i), 4.12 (2H, d, *J* = 15 Hz, N***CH₂***-i), 4.21 (2H, s, N***CH₂***-r), 6.55 (2H, s, Ar***H***-e), 6.66 (1H, s, Ar***H***-n), 6.85 (2H, s, Ar***H***-c), 6.93 (1H, s, Ar***H***-l).

^{11}B NMR (160.4 MHz, CD_2Cl_2) δ_B: 2.33

^{13}C{^{1}H} NMR (125.7 MHz, CD_2Cl_2) δ_C: 16.2 (***Me***-g), 16.6 (***Me***-p), 20.4 (***Me***-q), 20.5 (***Me***-h), 54.3 (***CH₂***-r), 55.4 (***CH₂***-i), 116.7 (***Ar***CH_2-f), 117.1 (***Ar***CH_2-o), 125.1 (***Ar***H-e), 125.9 (***Ar***-k), 127.5 (***Ar***-b), 128.7 (***Ar***-d), 129.3 (***Ar***-m), 131.2 (***Ar***H-c), 132.4 (***Ar***H-n), 133.2 (***Ar***H-l), 149.4 (***Ar***OB-a), 153.1 (***Ar***OH-j).

Aminobisphenolborate (4)

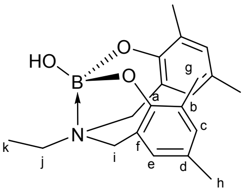

^{1}H NMR (500 MHz, CD_2Cl_2) δ_H: 1.17 (3H, t, *J* = 7 Hz, ***CH₃***-k) 2.18 (6H, s, ***Me***-h) 2.20 (6H, s, ***Me***-g) 3.16 (2H, q, *J* = 7 Hz, ***CH₂***-j) 3.93-4.02 (4H, m, NC***H₂***-i), 6.60 (2H, s, Ar***H***-e) 6.89 (2H, s, Ar***H***-c).

^{11}B NMR (160.4 MHz, CD_2Cl_2) δ_B: 2.40

^{13}C{^{1}H} NMR (125.7 MHz, CD_2Cl_2) δ_C: 5.4 (***CH₃***-k), 16.3 (***Me***-g), 20.3 (***Me***-h), 46.4 (***CH₂***-j), 52.3 (***CH₂***-i), 115.2 (***Ar***-f), 127.1 (***Ar***-b), 130.7 (***Ar***-d), 124.8 (***Ar***H-e), 131.1 (***Ar***H-c), 150.1 (***Ar***OB-a).

NMR Assignments of Compounds 6-9

Compound 6

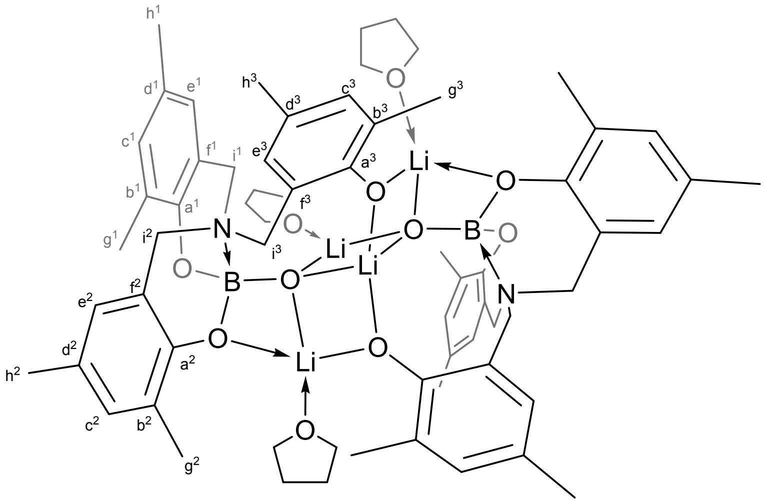

^{1}H NMR (500 MHz, d$_8$-THF) δ$_H$: 1.89 (3H, s, ***Me***-g^1), 2.08 (6H, s, ***Me***-h^1,h^3), 2.12 (3H, s, ***Me***-g^3), 2.17 (3H, s, ***Me***-h^2), 2.30 (3H, s, ***Me***-g^2), 3.29 (1H, d, *J* = 15 Hz, ArCH***H***N-i^{1a}), 3.38 (1H, d, *J* = 15 Hz, ArCH***H***N-i^{3a}), 3.73 (1H, d, *J* = 15 Hz, ArCH***H***N-i^{2a}), 4.07 (1H, d, *J* = 15 Hz, ArCH***H***N-i^{2b}), 5.03 (1H, d, *J* = 15 Hz, ArCH***H***N-i^{1b}), 5.08 (1H, d, *J* = 15 Hz, ArCH***H***N-i^{3b}), 6.43 (1H, s, Ar***H***-e^1), 6.60 (1H, s, Ar***H***-e^3), 6.61 (1H, s, Ar***H***-e^2), 6.65 (1H, s, Ar***H***-c^1), 6.75 (1H, s, Ar***H***-c^3), 6.81 (1H, s, Ar***H***-c^2).

^{7}Li NMR (194.4 MHz, d$_8$-THF, 258 K) δ$_{Li}$: 2.33 (1Li), 1.36 (2Li), 0.56 (1Li).

^{11}B NMR (160.4 MHz, d$_8$-THF) δ$_B$: 3.04

^{13}C{^{1}H} NMR (125.7 MHz, d$_8$-THF) δ$_C$: 16.2 (***Me***-g^1), 16.5 (***Me***-g^2), 18.4 (***Me***-g^3), 20.2 (***Me***-h^1), 20.4 (***Me***-h^2,h^3), 52.9 (***CH$_2$***-i^1), 53.7 (***CH$_2$***-i^3), 56.5 (***CH$_2$***-i^2), 118.7 (***Ar***CH$_2$-f^3), 118.9 (***Ar***CH$_2$-f^1), 119.6 (***Ar***Me-d^3), 119.9 (***Ar***CH$_2$-f^2), 125.8 (***Ar***H-d^1,e^1), 126.3 (***Ar***Me-b^3), 126.5 (***Ar***Me-b^2), 126.6 (***Ar***H-e^2), 126.8 (***Ar***Me-b^1), 127.7 (***Ar***Me-d^2), 130.0 (***Ar***H-c^1), 130.7 (***Ar***H-c^2), 131.3 (***Ar***H-e^3), 132.2 (***Ar***H-c^3), 151.8 (***Ar***OB-a^1), 152.4 (***Ar***OB-a^2), 166.2 (***Ar***OSn-a^3).

Compound 7

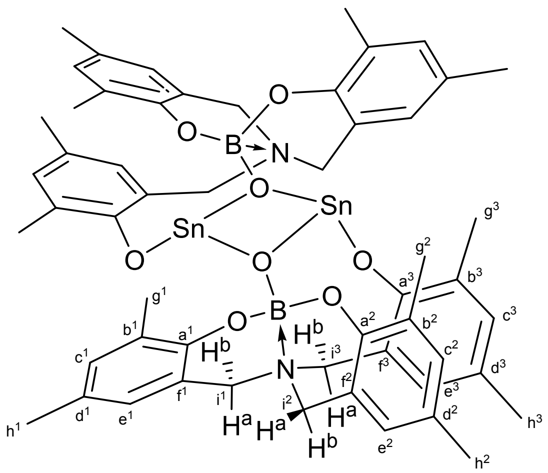

^{1}H NMR (500 MHz, C_6D_6) δ_H: 1.73 (3H, s, ***Me***-g^2), 1.90 (3H, s, ***Me***-h^3), 2.10 (3H, s, ***Me***-h^2), 2.26 (3H, s, ***Me***-h^1), 2.29 (3H, s, ***Me***-g^1), 2.32 (3H, s, ***Me***-g^3), 3.10 (1H, d, *J* = 15 Hz, ArCH***H***N-i^{3a}), 3.19 (1H, d, *J* = 15 Hz, ArCH***H***N-i^{2a}), 3.29 (1H, d, *J* = 15 Hz, ArCH***H***N-i^{1a}), 4.30 (1H, d, *J* = 15 Hz, ArCH***H***N-i^{2b}), 5.84 (1H, d, *J* = 15 Hz, ArCH***H***N-i^{3b}), 5.91 (1H, d, *J* = 15 Hz, ArCH***H***N-i^{1b}), 6.09 (1H, s, Ar***H***-e^2), 6.32 (1H, s, Ar***H***-e^3), 6.36 (1H, s, Ar***H***-c^2), 6.68 (1H, s, Ar***H***-c^3), 6.74 (1H, s, Ar***H***-e^1), 6.83 (1H, s, Ar***H***-c^1).

^{11}B NMR (160.4 MHz, C_6D_6) δ_B: 2.63

^{13}C{^{1}H} NMR (125.7 MHz, C_6D_6) δ_C: 16.6 (***Me***-g^1), 17.1 (***Me***-g^2), 18.6 (***Me***-g^3), 20.3 (***Me***-h^3), 20.5 (***Me***-h^2), 20.8 (***Me***-h^1), 57.4 (***CH₂***-i^2), 60.4 (***CH₂***-i^1), 61.8 (***CH₂***-i^3), 115.9 (***Ar***CH$_2$-f^1), 119.3 (***Ar***CH$_2$-f^2), 122.2 (***Ar***CH$_2$-f^3), 123.3 (***Ar***H-e^2), 124.8 (***Ar***Me-d^3), 124.9 (***Ar***H-e^1), 126.4 (***Ar***Me-b^2), 127.3 (***Ar***Me-b^3), 127.7 (***Ar***Me-d^2), 128.4 (***Ar***Me-b^1), 128.6 (***Ar***Me-d^1), 129.9 (***Ar***H-c^2), 131.7 (***Ar***H-c^1), 131.8 (***Ar***H-c^3), 132.2 (***Ar***H-e^3), 149.1 (***Ar***OB-a^2), 149.6 (***Ar***OB-a^1), 157.9 (***Ar***OSn-a^3).

^{119}Sn NMR (186.3 MHz, C_6D_6) δ_{Sn}: -412.3

Compound 8

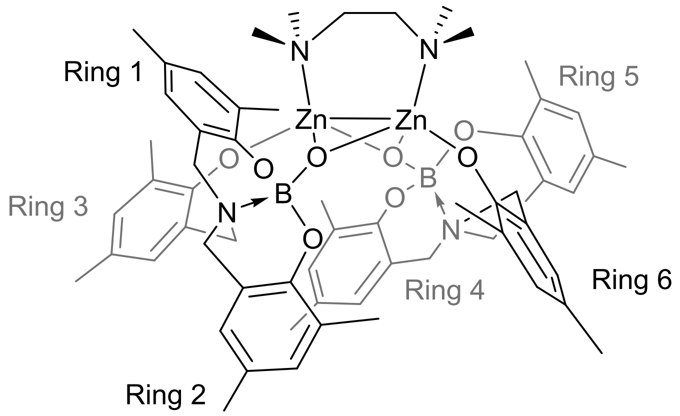

^{11}B NMR (160.4 MHz, C_6D_6) δ_B: 2.74

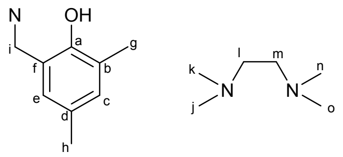

Table S1: Complete ^1H (500 MHz) and $^{13}C\{^1H\}$ (125.7 MHz) NMR Assignment of Compound 8 in C_6D_6.

Position	Ring 1		Ring 2		Ring 3		
	^1H	^{13}C	^1H	^{13}C	^1H	^{13}C	
a		152.6		150.4		161.7	
b		127.3		126.8		128.2	
c	6.88	131.1	6.74	130.77	7.11	132.8	1H, s
d		127		126.6		122.3	
e	6.39	125.6	5.87	124.8	6.64	130.5	1H, s
f		117.2		117.8		117.1	
g	2.23	16.6	2.14	16.3	2.51	18.8	3H, s
h	2.18	20.6	2.05	20.5	2.32	20.7	3H, s
i	3.40/3.97	57.8	3.27/4.25	53.4	2.90/3.33	51.8	1H, d, J = 15 Hz
**Position**	**Ring 4**		**Ring 5**		**Ring 6**		
	^1H	^{13}C	^1H	^{13}C	^1H	^{13}C	
a		153.1		149.4		163.0	
b		126.85		129.1		130.0	
c	6.93	130.8	6.13	131.2	7.19	133.2	1H, s
d		127.3		127.1		122.7	
e	6.43	126.5	6.19	125.1	6.83	130.4	1H, s
f		117.9		117.3		116.7	
g	2.37	17.1	1.97	16.4	2.62	18.4	3H, s
h	2.23	20.7	1.70	20.0	2.35	20.9	3H, s
i	3.60/4.32	58.5	3.52/4.81	55.2	3.87/5.07	52.3	1H, d, J = 15 Hz

Position	^1H		^{13}C
j	2.95	3H, s	48.0
k	1.99	3H, s	43.9
l	1.38/3.40	1H, m	56.4
m	1.16/2.51	1H, m	56.7
n	2.46	3H, s	47.9
o	1.89	3H, s	43.8

Compound 9

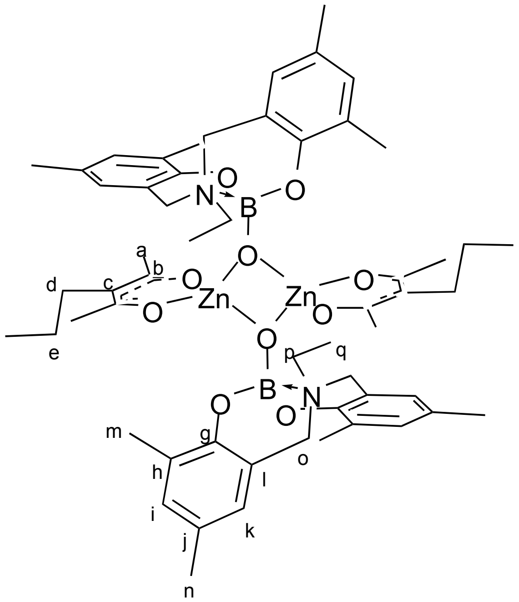

^{1}H NMR (500 MHz, C_6D_6) δ_H: 0.55 (3H, t, *J* = 7 Hz, ***CH$_3$***-q), 0.87 (3H, t, *J* = 7 Hz, ***CH$_3$***-f), 1.27 (2H, qt, *J* = 7,7 Hz, ***CH$_2$***-e), 1.85 (2H, t, *J* = 7 Hz, ***CH$_2$***-d), 1.92 (6H, s, ***Me***-a), 2.16 (6H, s, ***Me***-n), 2.26 (6H, s, ***Me***-m), 3.05 (2H, q, *J* = 7 Hz, ***CH$_2$***-p), 3.61 (4H, s, ***CH$_2$***-o), 6.27 (2H, s, Ar***H***-k), 6.70 (2H, s, Ar***H***-i).

^{11}B NMR (160.4 MHz, C_6D_6) δ_B: 2.82

^{13}C{^{1}H} NMR (125.7 MHz, C_6D_6) δ_C: 6.2 (***CH$_3$***-q), 14.6 (***CH$_3$***-f}), 16.8 (Ar***Me***-m), 20.8 (***Me***-n}), 24.2 (***CH$_2$***-e}), 26.9 (***CH$_3$***-a}), 33.6 (***CH$_2$***-d}), 46.7 (N***CH$_2$***-p), 52.7 (***CH$_2$***-o), 109.1 (***C***-c}), 116.5 (***Ar***CH$_2$-l), 125.0 (***Ar***H-k), 126.5 (**Ar**Me-j), 128.2 (***Ar***Me-h), 130.9 (***Ar***H-i}), 150.9 (***Ar***OB-g}), 191.6 (***C***O-b})

Supplementary Crystallographic Data

*Table S2: Crystal and structure refinement data for compounds **1**, **2**, **3** and **5**.*

Compound	1	2	3	5
Empirical formula	C_{35} H_{38} B Cl_6 N O_3	C_{26} H_{30} B N O_2	C_{126} H_{152} B_4 N_4 O_{19}	C_{22} H_{30} B N O_3
Formula weight	744.17	399.32	2069.75	367.28
Temperature	150.00(10) K	150.00(10) K	150.01(10) K	150.01(10) K
Wavelength	1.54184 Å	1.54184 Å	0.71073 Å	1.54184 Å
Crystal system	Triclinic	Orthorhombic	Monoclinic	Monoclinic
Space group	P-1	$Pca2_1$	C2/c	$P2_1/c$
Unit cell dimensions	a = 10.8014(2) Å	a = 18.6486(3) Å	a = 44.507(2) Å	a = 11.80335(17) Å
	b = 12.0164(3) Å	b = 14.9122(3) Å	b = 11.2530(2) Å	b = 14.3153(2) Å
	c = 14.4795(4) Å	c = 15.7148(3) Å	c = 32.5540(17) Å	c = 12.36797(19) Å
	a= 105.746(2)°.	α= 90°	α= 90°	α= 90°
	b= 95.6716(19)°.	β= 90°	β = 136.605(9)°.	β = 99.5679(14)°
	g = 90.670(2)°.	γ= 90°	γ= 90°	γ= 90°
Volume	1798.51(8) Å3	4370.14(14) $Å^3$	11201.4(15) $Å^3$	2060.73(5) $Å^3$
Z	2	8	4	4
Density (calculated)	1.374 Mg m^{-3}	1.214 Mg m^{-3}	1.227 Mg m^{-3}	1.184 Mg m^{-3}
Absorption coefficient	4.641 mm^{-1}	0.582 mm^{-1}	0.081 mm^{-1}	0.606 mm^{-1}
F(000)	772	1712	4432	792
Crystal size	0.400 x 0.200 x 0.150 mm^3	0.220 x 0.150 x 0.110 mm^3	0.535 x 0.347 x 0.200 mm^3	0.607 x 0.182 x 0.139 mm^3
Theta range for data collection	3.825 to 73.361°.	3.795 to 86.839°.	2.922 to 26.372°.	4.763 to 73.185°.
Index ranges	-13<=h<=12, -14<=k<=14, -17<=l<=17	-22<=h<=19, -19<=k<=19, -18<=l<=18	-55<=h<=54, -13<=k<=14, -40<=l<=40	-14<=h<=10, -17<=k<=17, -15<=l<=15
Reflections collected	19468	42251	48859	15994
Independent reflections	7185 [R(int) = 0.0312]	8569 [R(int) = 0.0491]	11430 [R(int) = 0.0281]	4098 [R(int) = 0.0384]
Completeness to theta = 67.684°	99.90%	99.30%	99.80%	100.00%
Absorption correction	Semi-empirical from equivalents	Semi-empirical from equivalents	Semi-empirical from equivalents	Semi-empirical from equivalents
Max. and min. transmission	1.00000 and 0.77849	1.00000 and 0.63085	1.00000 and 0.92023	1.00000 and 0.55905
Refinement method	Full-matrix least-squares on F^2	Full-matrix least-squares on F^2	Full-matrix least-squares on F^2	Full-matrix least-squares on F^2
Data / restraints / parameters	7185 / 36 / 481	8569 / 1 / 550	11430 / 182 / 848	4098 / 0 / 250
Goodness-of-fit on F^2	1.052	1.092	1.017	1.065
Final R indices [I>2σ(I)]	R1 = 0.0424, wR2 = 0.1151	R1 = 0.0731, wR2 = 0.1782	R1 = 0.0601, wR2 = 0.1566	R1 = 0.0483, wR2 = 0.1331
R indices (all data)	R1 = 0.0446, wR2 = 0.1173	R1 = 0.0763, wR2 = 0.1824	R1 = 0.0775, wR2 = 0.1695	R1 = 0.0541, wR2 = 0.1388
Absolute structure parameter		0.14(11)		
Extinction coefficient	n/a	n/a	n/a	n/a
Largest diff. peak and hole	0.455 and -0.458 e.Å-3	0.758 and -0.248 e.$Å^{-3}$	0.852 and -0.452 e.$Å^{-3}$	0.309 and -0.320 e.$Å^{-3}$
CCDC Number	2194565	2194566	2194572	2194567

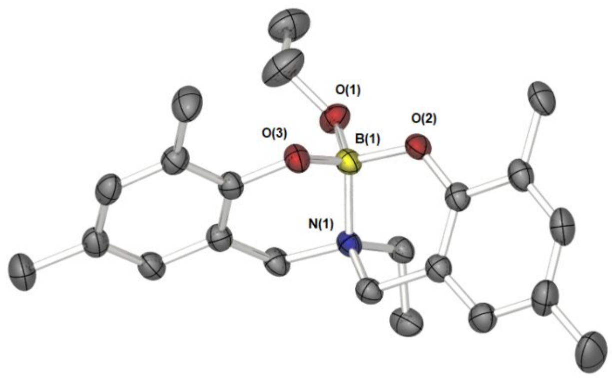

*Figure S1: The molecular structure of compound **5**. Thermal ellipsoids are shown at 50 % probability. Hydrogen atoms are omitted for clarity.*

*Table S3: Selected bond lengths and angles from compound **5**.*

**Bond Lengths (Å)**		**Bond Angles (˚)**	
B(1)-O(1)	1.417(2)	O(1)-B(1)-O(2)	110.57(13)
B(1)-O(2)	1.4569(19)	O(1)-B(1)-O(3)	116.31(13)
B(1)-O(3)	1.4533(19)	O(1)-B(1)-N(1)	106.04(11)
B(1)-N(1)	1.6522(19)	O(2)-B(1)-O(3)	108.84(12)
		O(2)-B(1)-N(1)	107.56(11)
		O(3)-B(1)-N(1)	107.11(11)

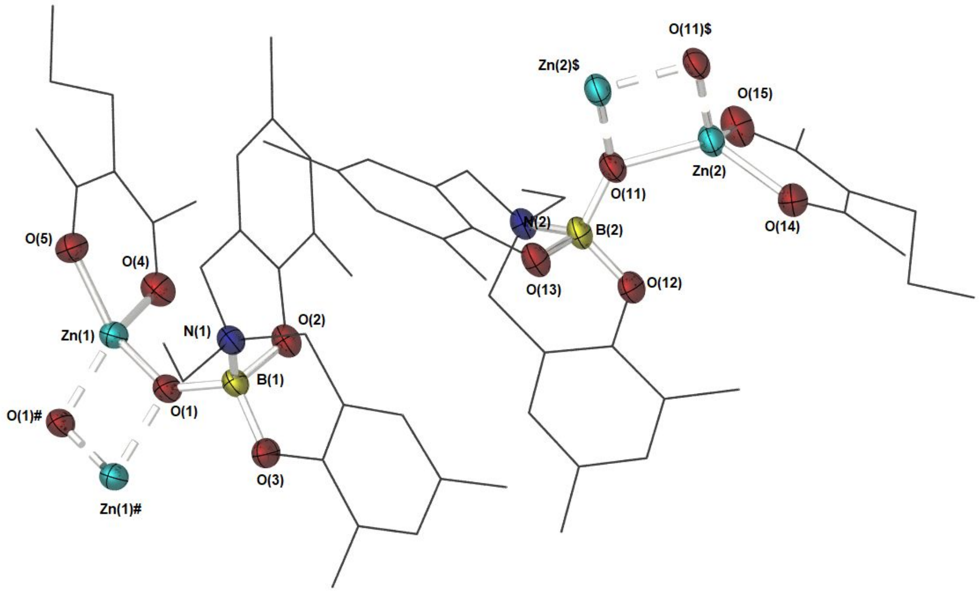

Figure S2: The asymmetric unit cell of compound ***9****. This comprises two halves of the dimeric molecular structure. Equivalent atoms that afford the full dimeric structure are generated by the symmetry operators: # = -X, 1-Y, -Z and $ = 1-X, 1-Y, 1-Z. Thermal ellipsoids are shown at 50 % probability. Hydrogen atoms and solvent present within the unit cell have been omitted for clarity.*

*Table S4: Crystal and structure refinement data for compounds **6**, **7**, **8**, and **9**.*

**Compound**	**6**	**7**	**8**	**9**
Empirical formula	$C_{78}H_{108}B_2Li_4N_2O_{14}$	$C_{69.40}H_{90.80}B_2N_2O_{11.85}Sn_2$	$C_{69}H_{85}B_2N_4O_8Zn_2$	$C_{59}H_{82}B_2Cl_6N_2O_{10}Zn_2$
Formula weight	1347.04	1401.64	1250.76	1344.32
Temperature	150.00(10) K	150.00(10) K	150.00(10) K	150(2) K
Wavelength	1.54184 Å	0.71073 Å	1.54184 Å	1.54184 Å
Crystal system	Monoclinic	Triclinic	Monoclinic	Monoclinic
Space group	I2/a	P-1	$P2_1/n$	$P2_1/c$
Unit cell dimensions	a = 16.4493(4) Å	a = 13.8134(6) Å	a = 16.3178(6) Å	a = 22.7416(3) Å
	b = 28.1416(6) Å	b = 14.3182(6) Å	b = 18.6726(3) Å	b = 17.76483(17) Å
	c = 21.2782(6) Å	c = 20.1271(7) Å	c = 21.2672(7) Å	c = 18.3446(2) Å
	a= 90°.	a= 82.865(3)°.	a= 90°.	a= 90°.
	b= 108.994(3)°.	b= 74.319(3)°.	b= 93.966(3)°.	b= 108.7628(14)°.
	g = 90°.	g = 61.353(4)°.	g = 90°.	g = 90°.
Volume	9313.6(4) $Å^3$	3363.4(3) $Å^3$	6464.5(3) $Å^3$	7017.38(15) $Å^3$
Z	4	2	4	4
Density (calculated)	0.961 Mg m^{-3}	1.384 Mg m^{-3}	1.285 Mg m^{-3}	1.272 Mg m^{-3}
Absorption coefficient	0.506 mm^{-1}	0.803 mm^{-1}	1.364 mm^{-1}	3.354 mm^{-1}
F(000)	2896	1452	2644	2808
Crystal size	0.380 x 0.150 x 0.110 mm^3	0.180 x 0.180 x 0.050 mm^3	0.380 x 0.100 x 0.030 mm^3	0.220 x 0.170 x 0.080 mm^3
Theta range for data collection	4.326 to 73.508°.	3.154 to 27.483°.	4.067 to 68.248°.	3.677 to 71.250°.
Index ranges	-16<=h<=20, -34<=k<=34, -26<=l<=26	-17<=h<=15, -18<=k<=16, -25<=l<=26	-19<=h<=18, -14<=k<=22, -25<=l<=25	-27<=h<=27, -21<=k<=21, -22<=l<=22
Reflections collected	34389	32529	34756	105694
Independent reflections	9206 [R(int) = 0.0365]	15386 [R(int) = 0.0302]	11819 [R(int) = 0.0602]	13506 [R(int) = 0.0447]
Completeness to theta = 67.684°	99.70%	99.70%	99.80%	100.00%
Absorption correction	Gaussian	Semi-empirical from equivalents	Semi-empirical from equivalents	
Max. and min. transmission	1.000 and 0.604	1.00000 and 0.82954	1.00000 and 0.71165	
Refinement method	Full-matrix least-squares on F^2	Full-matrix least-squares on F^2	Full-matrix least-squares on F^2	Full-matrix least-squares on F^2
Data / restraints / parameters	9206 / 0 / 491	15386 / 48 / 844	11819 / 37 / 800	13506 / 42 / 833
Goodness-of-fit on F^2	1.021	1.172	1.116	1.052
Final R indices [I>2σ (I)]	R1 = 0.0660, wR2 = 0.1897	R1 = 0.0524, wR2 = 0.1243	R1 = 0.0861, wR2 = 0.1956	R1 = 0.0483, wR2 = 0.1304
R indices (all data)	R1 = 0.0779, wR2 = 0.2019	R1 = 0.0671, wR2 = 0.1340	R1 = 0.1189, wR2 = 0.2126	R1 = 0.0569, wR2 = 0.1389
Extinction coefficient	n/a	n/a	n/a	n/a
Largest diff. peak and hole	0.462 and -0.262 e.$Å^{-3}$	1.709 and -1.297 e.$Å^{-3}$	0.712 and -0.492 e.$Å^{-3}$	0.749 and -0.438 e.$Å^{-3}$
CCDC Number	2194569	2194571	2194568	2194570

^{1}H, $^{13}C\{^{1}H\}$ and ^{1}H-DOSY NMR Spectra of Compounds 6-9

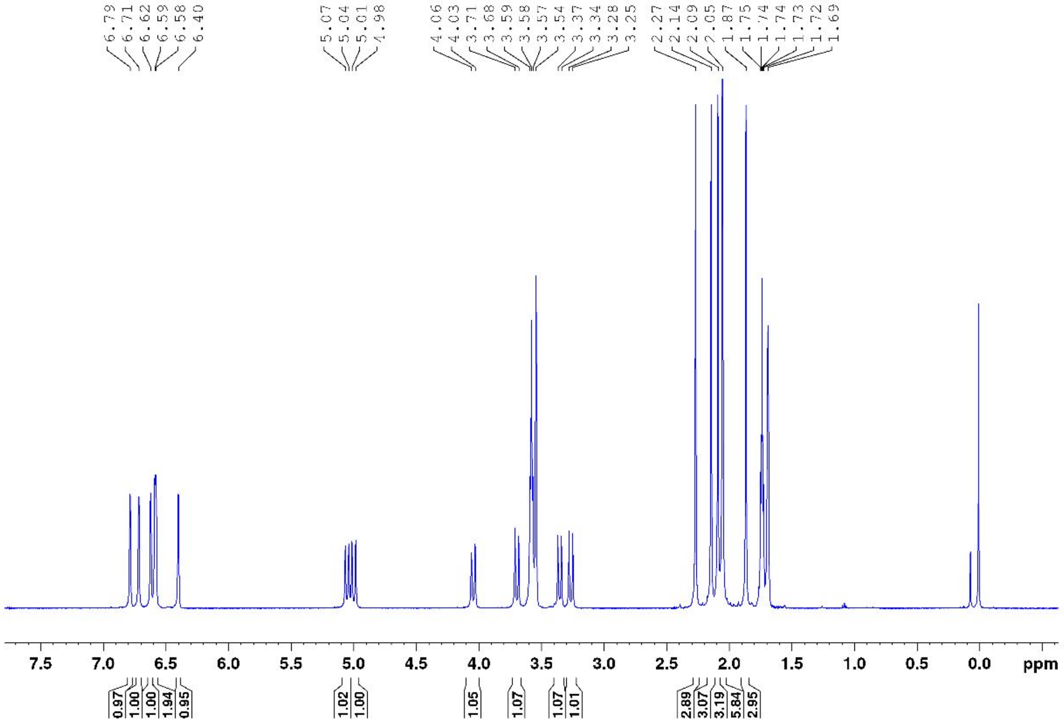

*Figure S3: ^{1}H NMR spectrum of compound **6** in d_8-THF.*

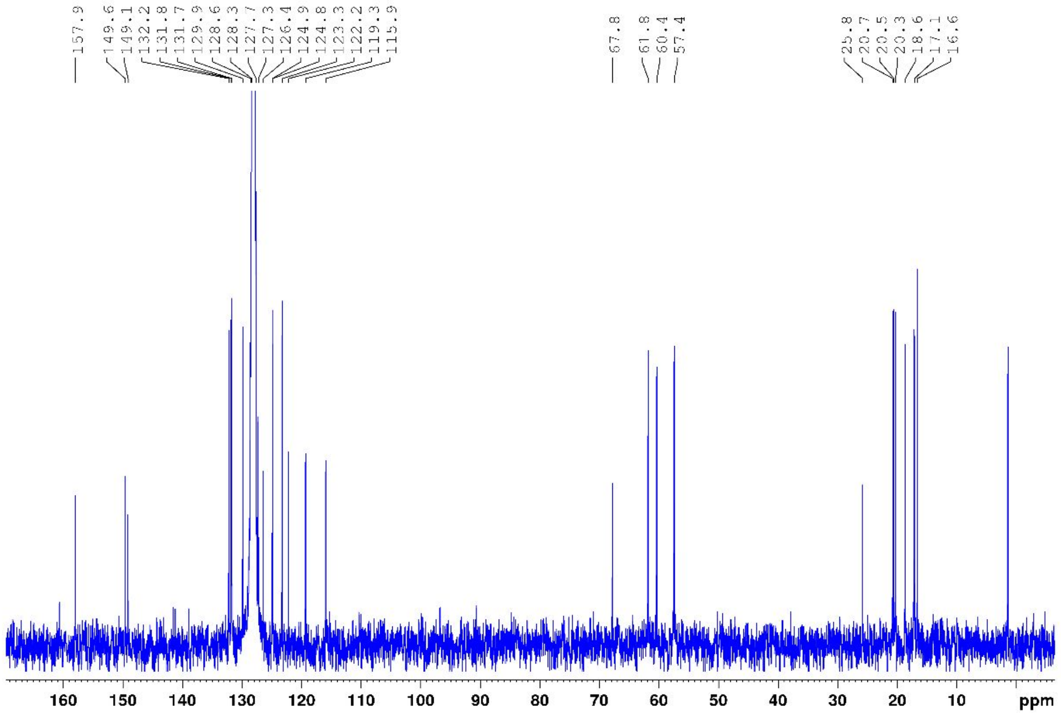

*Figure S4: $^{13}C\{^{1}H\}$ NMR spectrum of compound **6** in d_8-THF.*

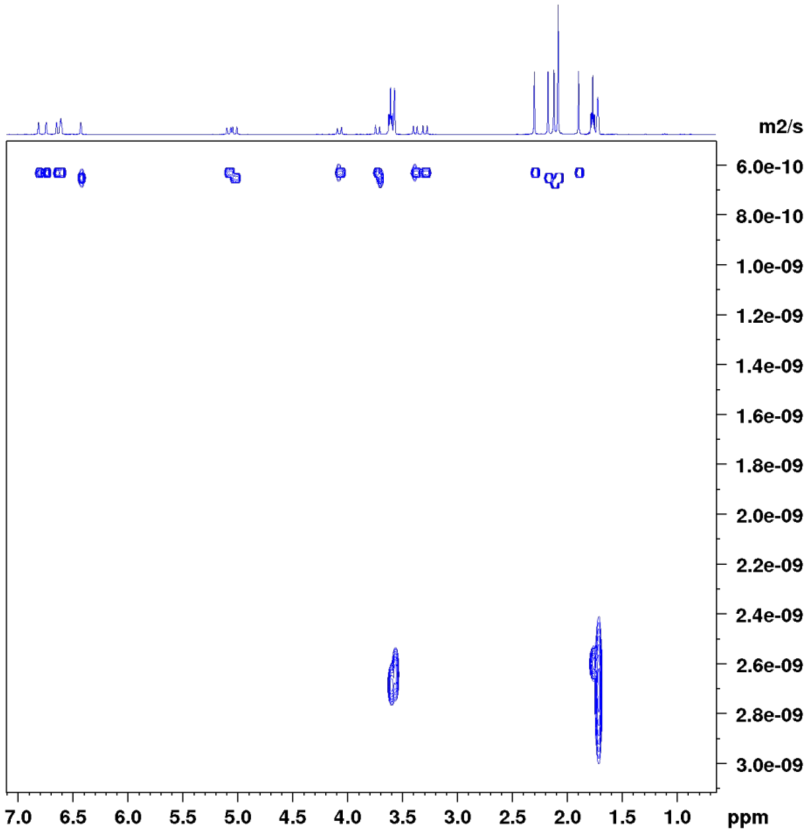

*Figure S5: ^{1}H-DOSY NMR spectrum of compound **6** in d_8-THF.*

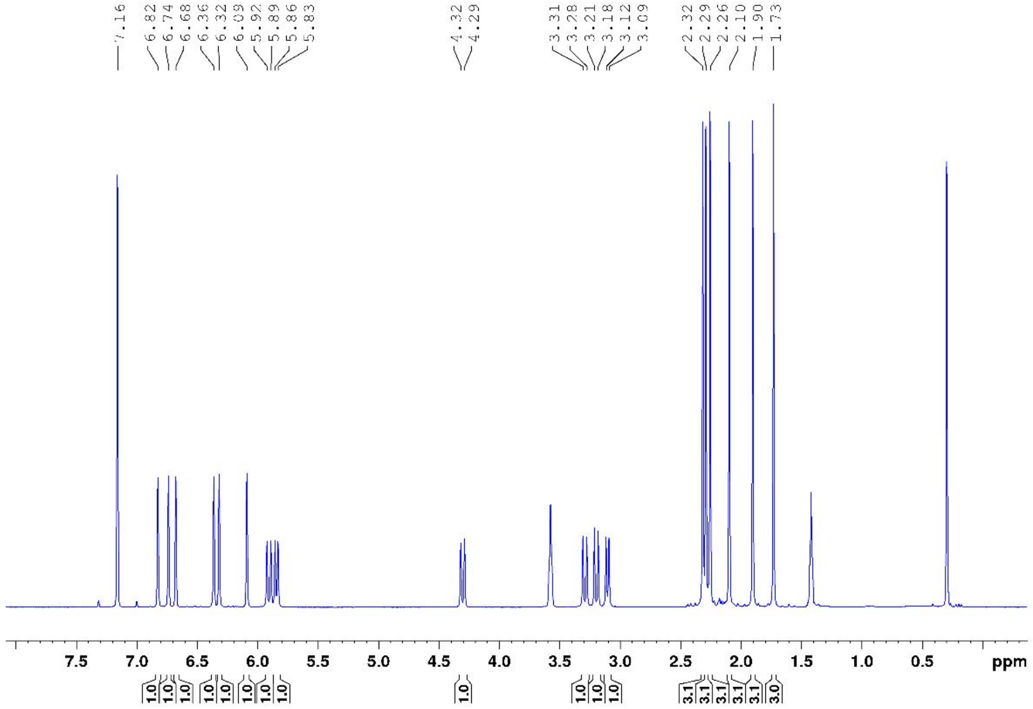

Figure S6: 1H NMR spectrum of compound 7 in C6D6.

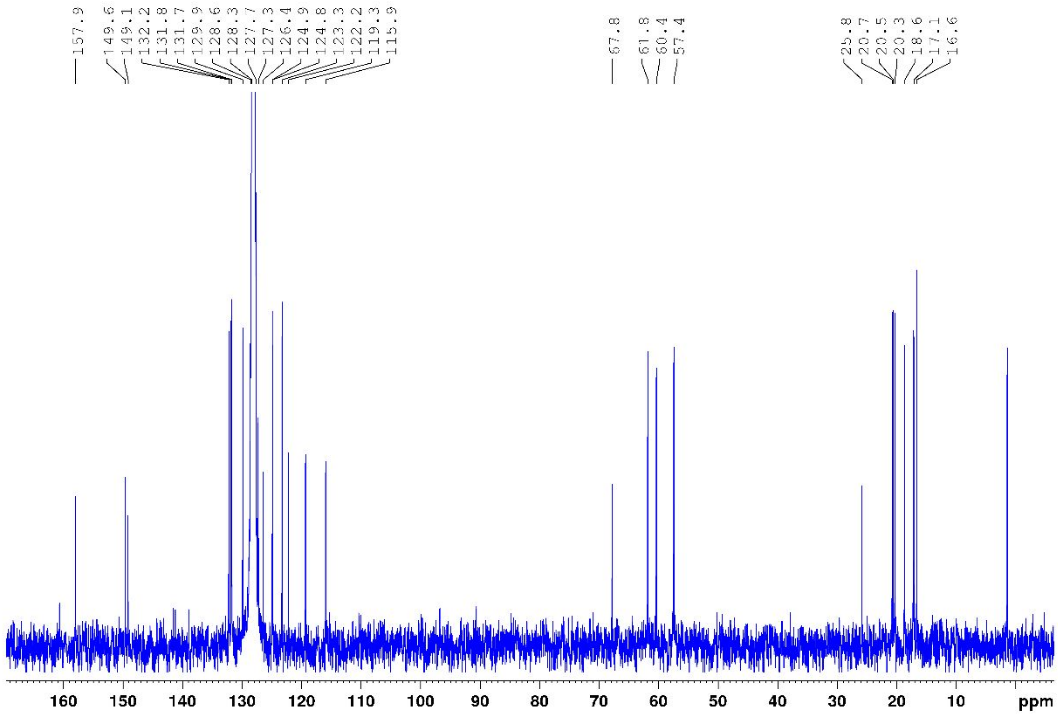

*Figure S7: ${}^{13}C\{{}^{1}H\}$ NMR spectrum of compound **7** in C_6D_6.*

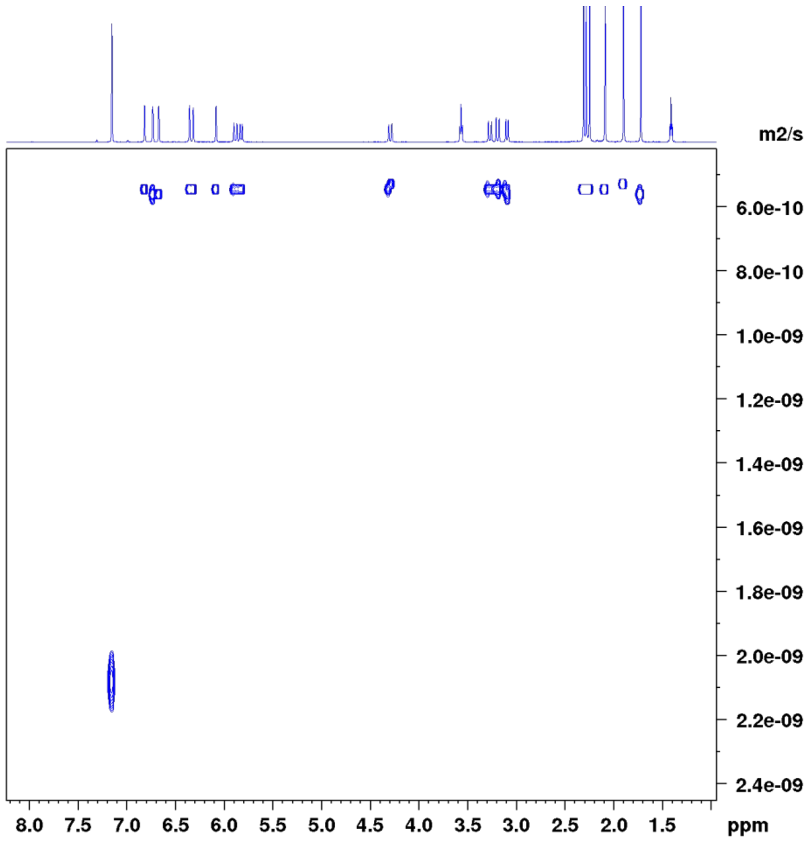

*Figure S8: ^{1}H-DOSY NMR spectrum of compound **7** in C_6D_6.*

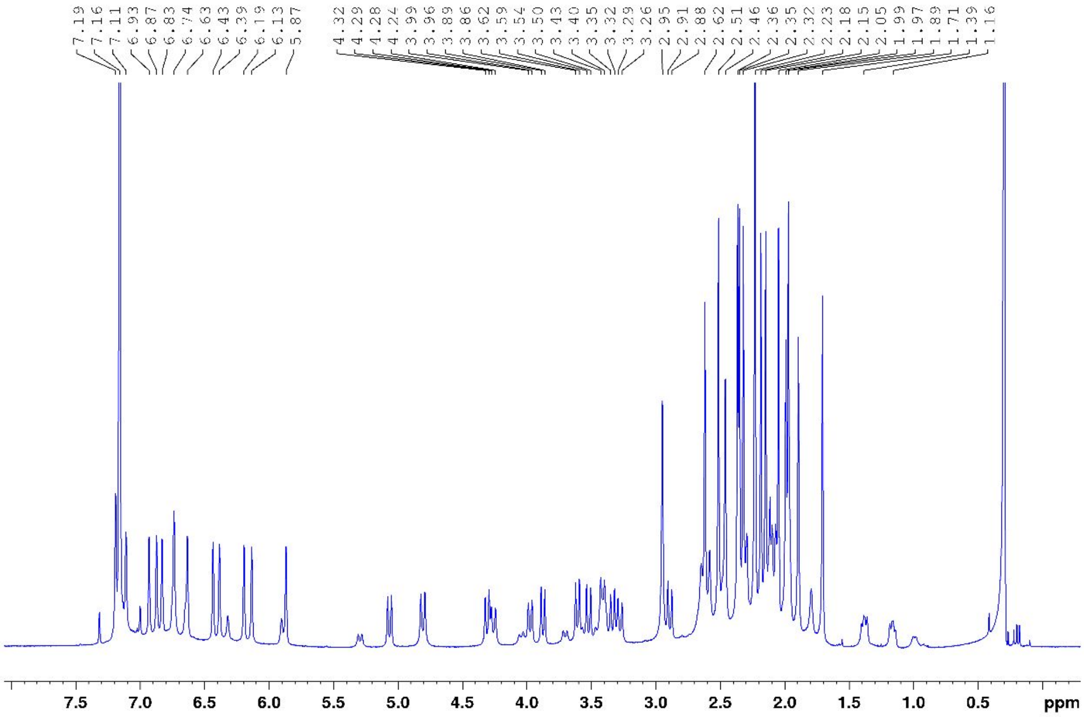

*Figure S9: ^{1}H NMR spectrum of compound **8** in C_6D_6.*

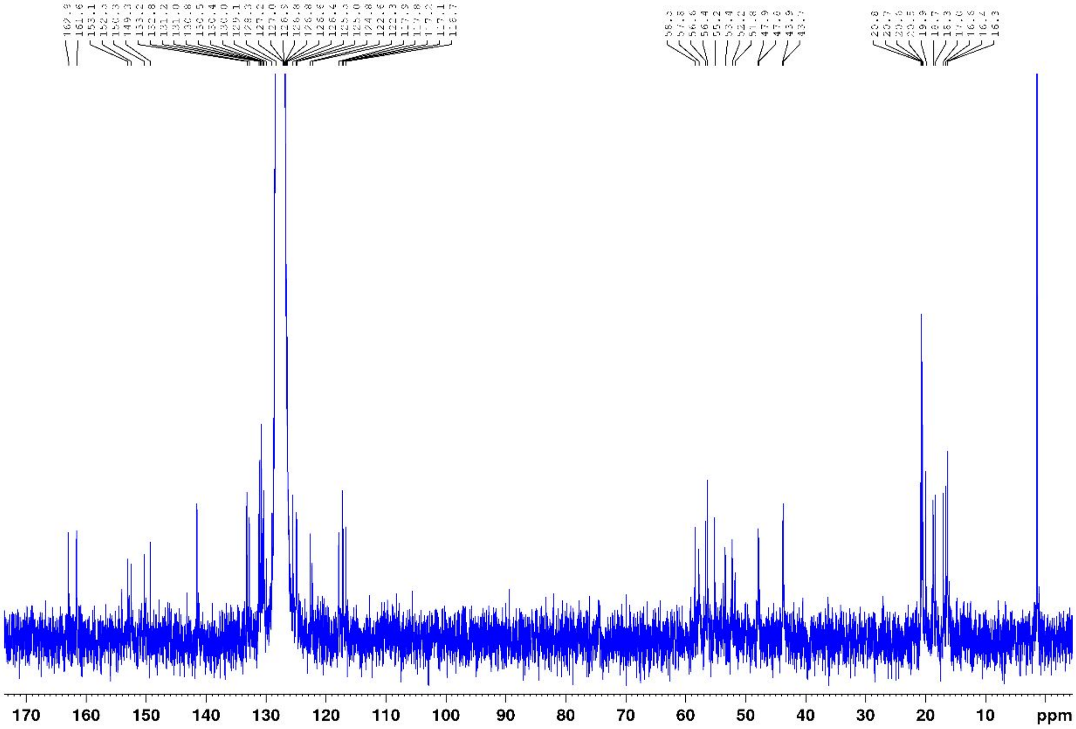

Figure S10: 13C{1H} NMR spectrum of compound 8 in C6D6.

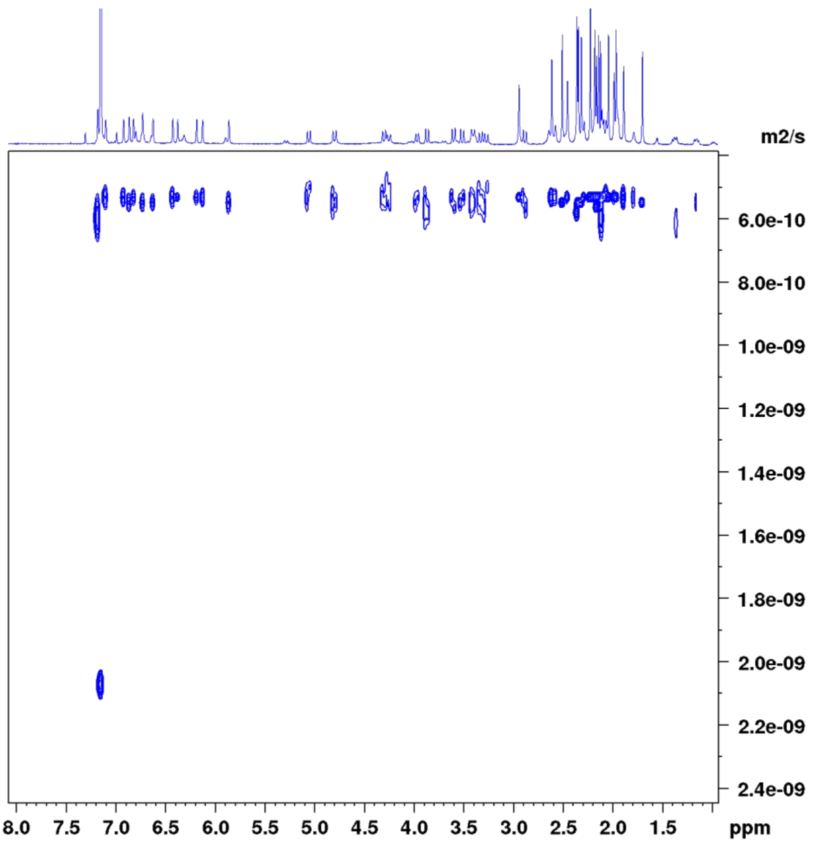

*Figure S11: ^{1}H-DOSY NMR Spectrum of Compound **8** in C_6D_6.*

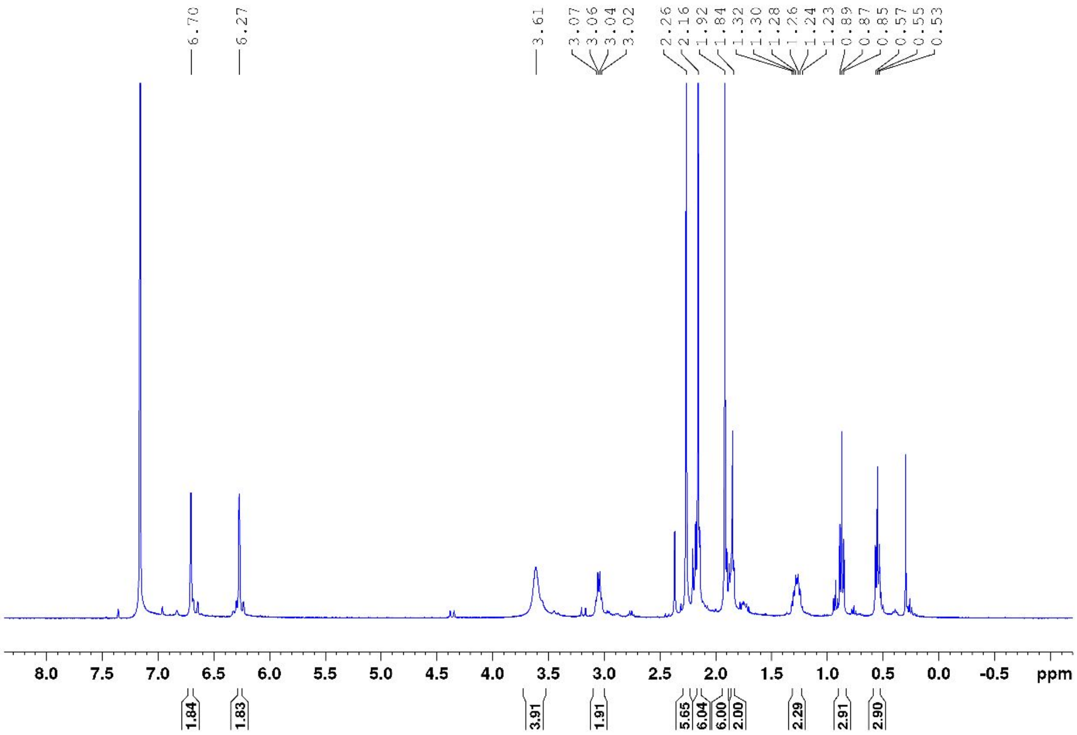

*Figure S12: ^{1}H NMR spectrum of compound **9** in C_6D_6.*

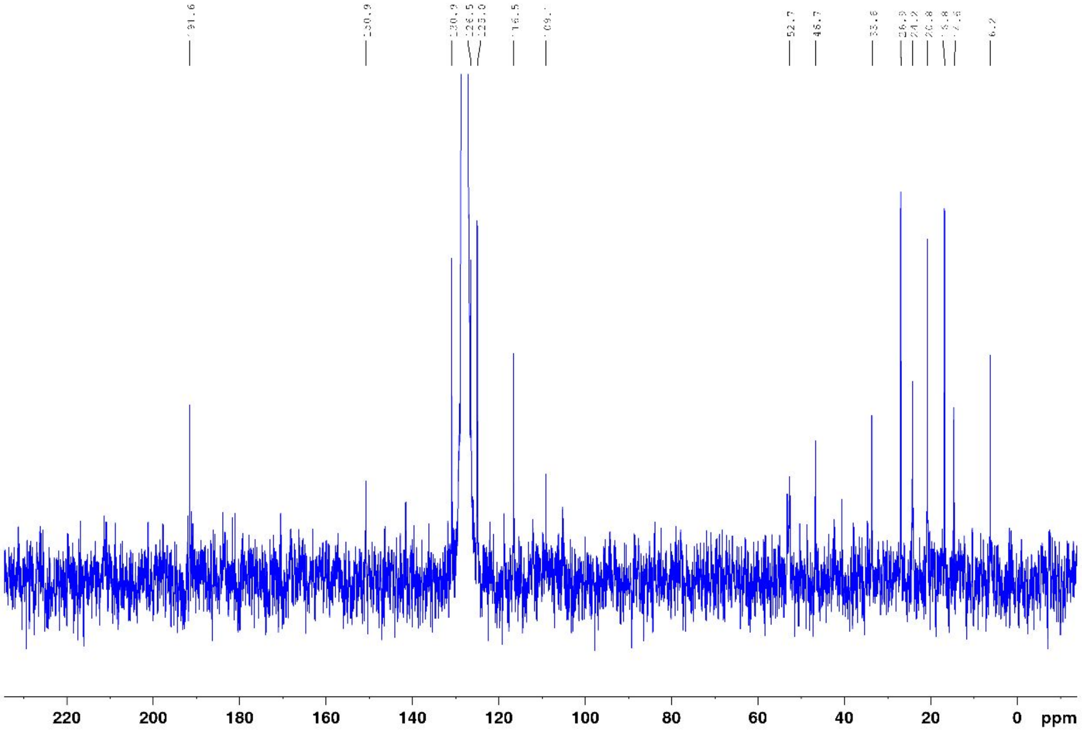

*Figure S13: $^{13}C\{^{1}H\}$ NMR spectrum of compound **9** in C_6D_6.*

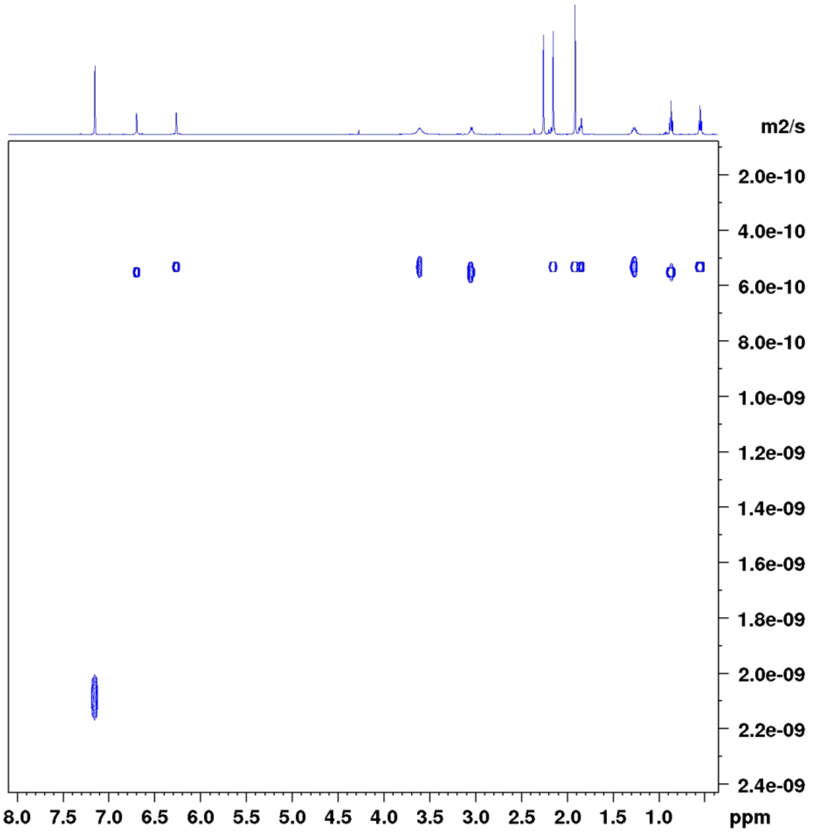

*Figure S14: ^{1}H-DOSY NMR Spectrum of Compound **9** in C_6D_6.*

DFT Studies

Cartesian Coordinates and Computed Energies (in Hartress)

I
SCF (BP86) Energy = -481.740975614
Enthalpy 0K = -481.648960
Enthalpy 298K = -481.640789
Free Energy 298K = -481.680811
Lowest Frequency = 106.5986 cm^{-1}
Second Frequency = 220.2629 cm^{-1}

B -2.10524 0.00060 0.00005
O -3.37706 0.00014 0.00008
O -1.17091 -1.18565 -0.00004
O -1.17130 1.18479 -0.00011
C 0.09388 0.71295 0.00003
C 0.09387 -0.71356 -0.00004
C 1.29468 -1.43033 -0.00000
C 2.51054 -0.70127 0.00002
C 2.51031 0.70205 0.00003
C 1.29408 1.43045 0.00001
H 1.28557 -2.52656 -0.00021
H 3.46341 -1.24624 0.00021
H 3.46307 1.24717 0.00008
H 1.28418 2.52668 -0.00007

II
SCF (BP86) Energy = -1082.63666996
Enthalpy 0K = -1082.233182
Enthalpy 298K = -1082.206659
Free Energy 298K = -1082.288256
Lowest Frequency = 26.2423 cm^{-1}
Second Frequency = 37.6269 cm^{-1}

B -0.04222 0.10328 -1.84324
N -0.07551 1.60069 -0.20875
O -0.52236 0.77887 -2.82874
O 1.40303 -0.23102 -1.67327
O -0.77927 -0.93407 -1.03370
C 2.10893 -0.34020 -0.53640
C 3.35496 -1.03782 -0.65495
C 4.20647 -1.15909 0.44908
H 5.15399 -1.70314 0.32496
C 3.88682 -0.60054 1.70452
C 2.67248 0.09581 1.80229
H 2.40135 0.56099 2.76181
C 1.77090 0.23500 0.72796
C 3.71242 -1.62460 -2.00014
H 2.93862 -2.33538 -2.34114
H 3.75618 -0.84073 -2.77742
H 4.68504 -2.14572 -1.96324
C 4.81069 -0.75595 2.89528
H 4.78347 -1.77999 3.31814
H 5.86518 -0.55222 2.63023
H 4.53359 -0.06343 3.70940
C 0.46431 0.97242 0.99166
H -0.30210 0.25867 1.34747
H 0.62218 1.69185 1.83382
C -1.94331 -0.69144 -0.41587
C -2.77583 -1.81785 -0.13342
C -3.97647 -1.63711 0.56543
H -4.60318 -2.51751 0.76888
C -4.39988 -0.36854 1.01599
C -3.55852 0.72414 0.74690
H -3.84885 1.72421 1.10109
C -2.34890 0.59836 0.03837
C -2.31987 -3.17924 -0.60210
H -1.35292 -3.45637 -0.14418
H -3.06307 -3.95751 -0.35565
H -2.14599 -3.18335 -1.69283
C -5.71575 -0.19245 1.74646
H -5.74739 0.76883 2.28912
H -6.58468 -0.20290 1.05830
H -5.88821 -0.99720 2.48519
C -1.52589 1.83444 -0.26276
H -1.86017 2.66215 0.40694
H -1.70547 2.12223 -1.31955
C 0.71477 2.70644 -0.76703
H 1.76978 2.38093 -0.76834
H 0.40269 2.77783 -1.82648
C 0.59551 4.05737 -0.03402
H -0.43354 4.45603 -0.06592
H 0.89385 3.97862 1.02744
H 1.25502 4.80654 -0.50833

III
SCF (BP86) Energy = -730.002041920
Enthalpy 0K = -729.823069
Enthalpy 298K = -729.809343
Free Energy 298K = -729.863160
Lowest Frequency = 32.4873 cm^{-1}
Second Frequency = 35.2845 cm^{-1}

C 3.31615 -1.20516 -0.83730
H 3.75903 -2.17323 -1.09543
C -2.74244 -1.42559 -0.37996
H -2.73529 -2.52204 -0.37285
C 3.96593 0.00155 -1.16228
H 4.93839 0.00225 -1.66566
C -3.79980 -0.70025 -0.98625
H -4.62436 -1.24688 -1.46166
B 0.28692 -0.00230 1.27976
O 0.96027 -0.00317 2.41788
O -0.61540 -1.21103 0.85284
O -0.61547 1.20986 0.85494
C -1.69357 0.71602 0.21982
N 1.47316 -0.00029 0.15670
C 3.31579 1.20731 -0.83457
H 3.75824 2.17611 -1.09068
C 2.07645 -1.17069 -0.20009
H 1.50657 -2.06359 0.06950
C 2.07608 1.17109 -0.19748
H 1.50588 2.06319 0.07419
C -1.69365 -0.71600 0.21833
C -2.74271 1.42642 -0.37720

H	-2.73588	2.52286	-0.36805
C	-3.79988	0.70200	-0.98487
H	-4.62456	1.24941	-1.45921

IV
SCF (BP86) Energy = -1082.62933465
Enthalpy 0K = -1082.225107
Enthalpy 298K = -1082.199069
Free Energy 298K = -1082.279669
Lowest Frequency = 26.5860 cm^{-1}
Second Frequency = 32.6133 cm^{-1}

B	-0.04850	0.29808	-1.64920
N	-0.05453	1.38929	-0.38660
O	-0.46829	0.86427	-2.75407
O	1.39616	-0.30055	-1.61179
O	-0.86193	-0.91742	-1.02646
C	2.15617	-0.38549	-0.52355
C	3.42686	-1.03880	-0.64278
C	4.28167	-1.10457	0.46334
H	5.25032	-1.61263	0.34694
C	3.94836	-0.53725	1.71458
C	2.70551	0.10982	1.81610
H	2.41707	0.57079	2.77259
C	1.80767	0.18629	0.73619
C	3.79262	-1.63117	-1.98202
H	3.04523	-2.37996	-2.30056
H	3.78841	-0.85816	-2.77160
H	4.78774	-2.10854	-1.95342
C	4.89066	-0.63433	2.89701
H	4.96658	-1.66669	3.29301
H	5.91981	-0.32150	2.63634
H	4.55259	0.00674	3.73014
C	0.45670	0.83796	0.90998
H	-0.29910	0.10869	1.24974
H	0.49891	1.63556	1.68074
C	-2.01583	-0.68547	-0.41074
C	-2.88977	-1.78523	-0.13405
C	-4.08012	-1.56044	0.56822
H	-4.73758	-2.41850	0.77205
C	-4.46128	-0.27879	1.02834
C	-3.58937	0.79026	0.75782
H	-3.85072	1.79945	1.10984
C	-2.39140	0.61378	0.04428
C	-2.47758	-3.15660	-0.61119
H	-1.52279	-3.46943	-0.14992
H	-3.24717	-3.91326	-0.37830
H	-2.29390	-3.15422	-1.70045
C	-5.76579	-0.06788	1.76962
H	-5.79367	0.92366	2.25528
H	-6.64765	-0.12396	1.10009
H	-5.92211	-0.82717	2.55927
C	-1.50735	1.78633	-0.29811
H	-1.64084	2.60460	0.43604
H	-1.71436	2.13167	-1.33105
C	0.76969	2.55896	-0.86814
H	1.81309	2.20631	-0.88111
H	0.43881	2.66049	-1.91915
C	0.66371	3.85153	-0.05297
H	-0.33968	4.30679	-0.10254
H	0.92509	3.71299	1.01230
H	1.37586	4.58705	-0.46703

V
SCF (BP86) Energy = -799.345404971
Enthalpy 0K = -799.003818
Enthalpy 298K = -798.980576
Free Energy 298K = -799.056397
Lowest Frequency = 26.4191 cm^{-1}
Second Frequency = 32.5596 cm^{-1}

O	-0.00396	2.59766	0.02067
B	-0.00107	1.28991	0.00969
C	3.99003	-0.82854	-0.28906
C	2.91820	-1.19280	-1.12007
H	3.06985	-1.97992	-1.87434
C	1.65428	-0.57277	-1.01835
C	3.76650	0.20085	0.64399
H	4.59261	0.52154	1.29682
C	2.51850	0.84217	0.75772
C	1.41551	0.46024	-0.06603
C	5.33286	-1.52590	-0.38268
H	5.47276	-2.26957	0.42687
H	5.44062	-2.06599	-1.33999
H	6.17228	-0.81076	-0.30338
C	2.35837	1.96214	1.77049
H	3.33943	2.33678	2.11615
H	1.75740	2.76796	1.31478
H	1.80063	1.61901	2.66441
C	0.56121	-1.02364	-1.97496
H	-0.29206	-1.47726	-1.44179
H	0.14673	-0.17203	-2.54343
H	0.94847	-1.76115	-2.70151
C	-3.98655	-0.84420	0.27102
C	-2.91337	-1.22184	1.09458
H	-3.06115	-2.02740	1.83001
C	-1.65195	-0.59520	1.00590
C	-1.41574	0.45566	0.07235
C	-2.51773	0.84559	-0.74858
C	-3.76341	0.19721	-0.64840
H	-4.58693	0.51903	-1.30395
C	-5.34525	-1.50512	0.39519
H	-5.26867	-2.49846	0.87221
H	-6.04590	-0.90483	1.00899
H	-5.82515	-1.64099	-0.59135
C	-2.35695	1.97580	-1.74979
H	-3.33768	2.35128	-2.09547
H	-1.76052	2.77873	-1.28316
H	-1.79445	1.64377	-2.64491
C	-0.55569	-1.06498	1.94965
H	-0.14022	-0.22459	2.53386
H	-0.94038	-1.81716	2.66240
H	0.29648	-1.50698	1.40509
